# Phosphoinositide regulates dynamic movement of the S4 voltage sensor in the second repeat in two-pore channel 3

**DOI:** 10.1016/j.jbc.2021.101425

**Published:** 2021-11-18

**Authors:** Kiichi Hirazawa, Michihiro Tateyama, Yoshihiro Kubo, Takushi Shimomura

**Affiliations:** 1 Division of Biophysics and Neurobiology, National Institute for Physiological Sciences, Okazaki, Japan; 2 Department of Physiological Sciences, The Graduate University for Advanced Studies, Hayama, Japan

**Keywords:** sodium channel, phosphoinositide, electrophysiology, ligand-binding protein, gating, two-pore channel, TPC, voltage sensor, voltage-gated cation channel, dynamic structure, ΔF-V relationship, F change-voltage relationship, Anap, 3-(6-acetylnaphthalen-2-ylamino)-2-aminopropanoic acid, CiVSP, *Ciona intestinalis* voltage sensitive phosphatase, cRNA, complimentary ribonucleic acid, F change, change of fluorescence intensity, G-V relationship, conductance-voltage relationship, INPP4B, inositol polyphosphate 4-phosphatase type II, MTSES, 2-Sulfonatoethyl methanethiosulfonate sodium salt, Nav, voltage-gated Na^+^ channel, NMDG, *N*-Methyl _D_-glucamine, PI(3,4)P_2_, phosphatidylinositol (3,4) bisphosphate, Q-V relationship, gating charge-voltage relationship, S4, the fourth helix, S6, the sixth helix, TM, transmembrane, TPC, Two-pore channel, V_1/2_, membrane voltage for half maximum activation, VCF, voltage clamp fluorometry, VSD, voltage sensor domain, VSP, voltage sensitive phosphatase, XtTPC3, *Xenopus tropicalis* TPC3.

## Abstract

The two-pore channels (TPCs) are voltage-gated cation channels consisting of single polypeptides with two repeats of a canonical 6-transmembrane unit. TPCs are known to be regulated by various physiological signals such as membrane voltage and phosphoinositide (PI). The fourth helix in the second repeat (second S4) plays a major role in detecting membrane voltage, whereas the first repeat contains a PI binding site. Therefore, each of these stimuli is detected by a unique repeat to regulate the gating of the TPC central pore. How these various stimuli regulate the dynamic structural rearrangement of the TPC molecule remain unknown. Here, we found that PI binding to the first repeat in TPC3 regulates the movement of the distally located second S4 helix, showing that the PI-binding signal is not confined to the pore gate but also transmitted to the voltage sensor. Using voltage clamp fluorometry, measurement of gating charges, and Cys-accessibility analysis, we observed that PI binding significantly potentiates the voltage dependence of the movement of the second S4 helix. Notably, voltage clamp fluorometry analysis revealed that the voltage-dependent movement of the second S4 helix occurred in two phases, of which the second phase corresponds to the transfer of the gating charges. This movement was observed in the voltage range where gate-opening occurs and was potentiated by PI. In conclusion, this regulation of the second S4 helix by PI indicates a tight inter-repeat coupling within TPC3, a feature which might be conserved among TPC family members to integrate various physiological signals.

Voltage-gated cation channels are membrane proteins, which permeate specific ions in response to the change of the membrane voltage (1). Their molecular structure features a unit of six transmembrane helices (6TM) (2). The N-terminal 4 helices form a voltage sensor domain (VSD), whereas the C-terminal 2 helices form a pore domain. The fourth helix (S4) in VSD has multiple positively charged residues (Arg and Lys). These positively charged residues enable S4 to move voltage dependently to gate the pore through the linker between S4 and S5 (3, 4, 5, 6). Two-pore channels (TPCs) have a specific feature that their single polypeptides have two repeats of 6TMs (7, 8), whereas voltage-gated K^+^ channels have one repeat of 6TM (9) and eukaryotic voltage-gated Na^+^ and Ca^2+^ channels (Navs) have four repeats of 6TMs within their single polypeptides (10, 11). Four 6TMs are assembled to make one minimal functioning channel.

TPC family has three members, TPC1–3. TPC family protein was initially identified as a Ca^2+^ release channel, which is located in endo/lysosome and activated by nicotinic acid adenine dinucleotide phosphate(8). It was reported that TPC1 and TPC2 are important for the maintenance of the ionic homeostasis of the intracellular organelle (8), autophagy (12, 13), nutrient sensing (14), and Ebola virus infection (15). Phosphatidylinositol (3,5) bisphosphate, a kind of phospholipid localized on the endo/lysosomal membrane, is an activator for TPC1,2 (16, 17). TPC1 is also regulated by membrane voltage in contrast to the lack of clear voltage dependence in TPC2 (18, 19, 20). Furthermore, TPC1 and TPC2 are reported to be regulated by mammalian target of rapamycin (14, 21), H^+^ (18, 22), Ca^2+^ (22), Mg^2+^ (17), and protein kinases (17). These findings indicate that TPC integrates multiple physiological signals.

In contrast to TPC1,2, there are limited reports about TPC3. As to the physiological function of TPC3, it is expressed in oocytes and fertilized eggs and is important for fertilization (23). It was also revealed that TPC3 is localized not only on the intracellular membrane, but also on the plasma membrane. TPC3 was previously reported as a simple voltage-gated Na^+^ channel with no sensitivity to phosphoinositide (PI) using mammalian expression system (24). However, we recently reported that TPC3 from *Xenopus tropicalis* (XtTPC3) is modulated by phosphatidylinositol (3,4) bisphosphate (PI(3,4)P_2_) and phosphatidylinositol (3,5) bisphosphate in *Xenopus* oocytes, showing that TPC3 also integrates multiple signals (25). Therefore, TPC3, as a representative of the TPC family with both PI and voltage sensitivity, is an ideal target to investigate how TPCs integrate multiple signals. When PI(3,4)P_2_ binds to TPC3, its voltage dependence is potentiated, allowing the pore to open at less depolarized membrane voltages. The binding site of PI(3,4)P_2_ is located in the former repeat (the first repeat) of two 6TMs within a monomer of TPC3, involving the N-terminal region, the first S4–S5 linker and the first S6 (25). In contrast, the voltage sensing is governed by the VSD in the second repeat (the second VSD), in which the S4 helix in the second VSD (second S4) plays a primary role (26). Therefore, it is suggested that the signal of the PI(3,4)P_2_ binding to the first repeat is transmitted to the second repeat. We previously reported that an inter-repeat interaction between the first and the second repeats within the same subunit is important for this transmission (25). The inter-repeat interaction site is located between the first S6 and the second S6 in the pore region of TPC3, involving hydrophilic and electrostatic interactions of Tyr293 (first S6), Arg297 (first S6), and Glu665 (second S6). Disruption of this interaction impairs the transmission of the signal of the PI(3,4)P_2_ binding, resulting in a largely weakened potentiation of the voltage dependence of TPC3 by PI(3,4)P_2_ (25). This inter-repeat transmission must affect the dynamic structural rearrangement of the second repeat. Although the structures of TPC1 with and without bound PI have been solved (19), the effect of the PI(3,4)P_2_ binding on the structural rearrangement of the second repeat remains unknown because of the lack of information of the dynamic structure of TPCs, hampering the understanding of the molecular basis of the regulation of TPC3.

To investigate how the PI(3,4)P_2_ binding to the first repeat affects the second repeat, we examined the dynamic structural rearrangement of the second S4, which governs the voltage-dependent gating of TPC3, by Cys-accessibility analysis, voltage clamp fluorometry (VCF) and measurement of the gating charges. The results obtained by these three methods demonstrated that the movement of the second S4 is potentiated by the PI(3,4)P_2_ binding to the first repeat. Therefore, it was shown that the signal of the PI(3,4)P_2_ binding is transmitted to the distally located second S4, revealing tight coupling across the whole molecule of TPC3.

## Results

**Figure 1:**
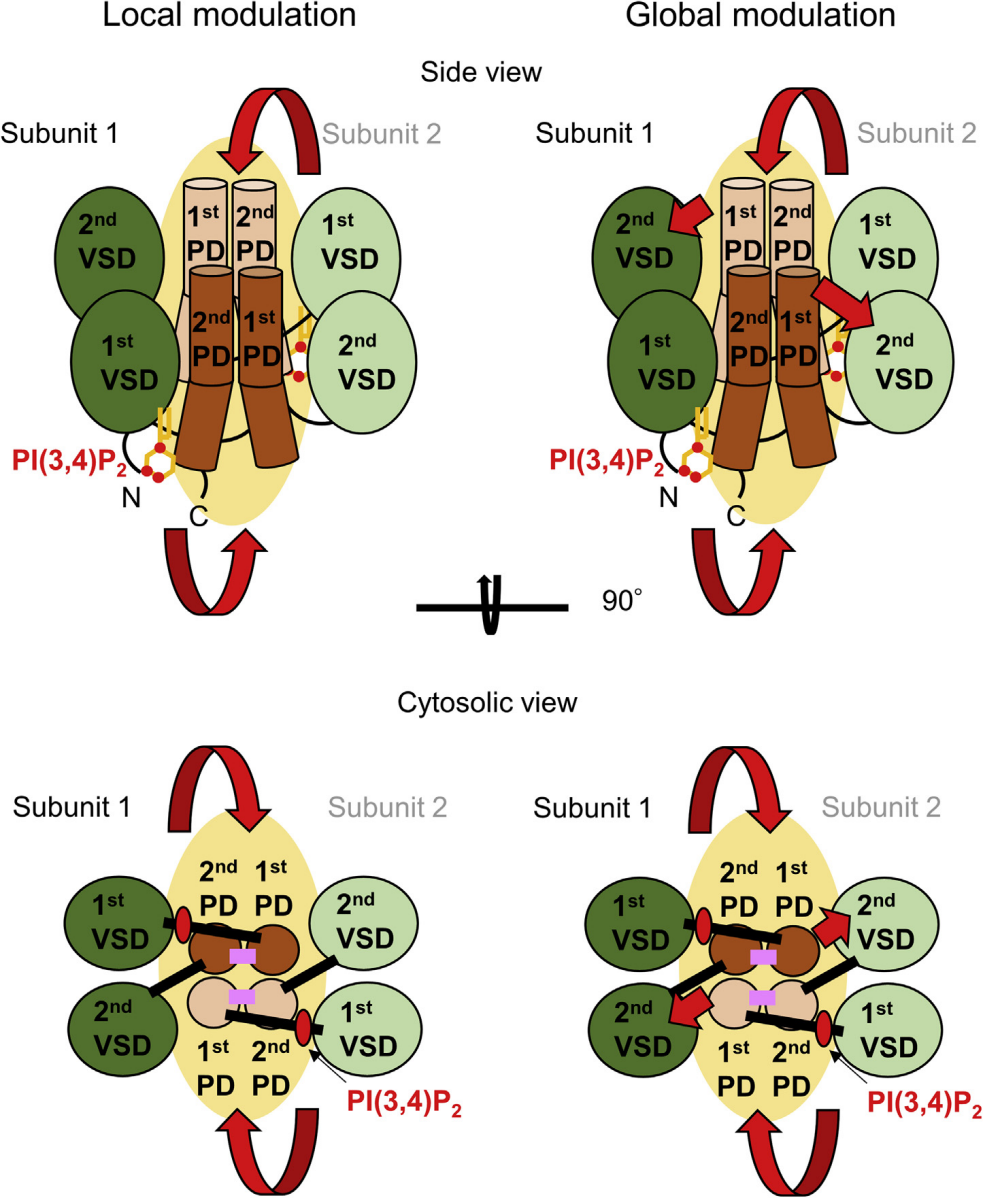
**Schematic presentation of the two hypothetical models.** Molecular structure of TPC3 homodimer and PI(3,4)P_2_ are schematically presented based on the high resolution structure of *Danio rerio* TPC3 (PDBID: 6V1Q) (26). Two subunits in the homodimer are drawn in *dark* and *pale colors*, respectively. *Upper panels*, side view of TPC3. The flow of the signal induced by PI(3,4)P_2_ binding is indicated by *red arrows*. The target domain of this signal transmission could be located in the same and/or another subunit compared with the PI(3,4)P_2_ binding site, and thus the PDs from both subunits are highlighted by *yellow ovals*, showing the ambiguity of the pathway of the signal transmission. In ‘local modulation’ (*left*), the effect of PI(3,4)P_2_ binding is confined around the pore domain, and thus the gate-opening step is potentiated without any effect on the second VSD. In ‘global modulation’ (*right*), the voltage-dependent structural rearrangement of the second VSD, especially the movement of the second S4 which governs the gate-opening, is also remotely modulated by PI(3,4)P_2_ binding to the first repeat. *Lower panels*, bottom-up view of TPC3 from the intracellular side. PI(3,4)P_2_ is depicted by *red ovals*. The intra-subunit interaction between the first and the second PD, which is critical for the potentiation of the voltage-dependent gating by PI(3,4)P_2_ (25) is depicted by *pink bars*. PD, pore domain; S4, the fourth helix; TPC3, two-pore channel 3; VSD, voltage sensor domain.

The aim of this study is to reveal how PI(3,4)P_2_ binding to the first repeat potentiates the voltage-dependent gating of TPC3. Voltage-dependent opening of the gate is generally composed of sequential steps (3, 6, 27). The first step is the movement of S4 in response to depolarization, and the second step is the cooperative gate-opening following the S4 movement. Therefore, in TPC3, the potentiation of the voltage-dependent gating by PI(3,4)P_2_ can be achieved through either of the following two possibilities (Fig. 1): one possibility is a direct modulation of the second gate-opening step without any effect on the second S4 movement. Another possibility is a modulation involving the second S4 movement. Here, the modulation in the former possibility is named as ‘local modulation’ of the pore gate (first S6 and second S6) by PI(3,4)P_2_, because the PI(3,4)P_2_-binding site involving the first S6 is located near the pore gate (25). In this scenario, the signal of the PI(3,4)P_2_ binding is transmitted to the second S6 *via* the inter-repeat interaction but does not spread to any other domain (Fig. 1). The modulation in the latter possibility is named as ‘global modulation’ because in this modulation, the PI(3,4)P_2_-binding signal is transmitted not only to the second S6, but also to the second VSD including the second S4, which is located far away from the PI(3,4)P_2_-binding site (Fig. 1). To examine the possible presence of ‘global modulation’, we investigated whether the PI(3,4)P_2_ binding affects the movement of the second S4 by Cys-accessibility analysis, VCF, and measurement of the gating charges.

### Validation of D511C-TPC3 for Cys-accessibility analysis

**Figure 2:**
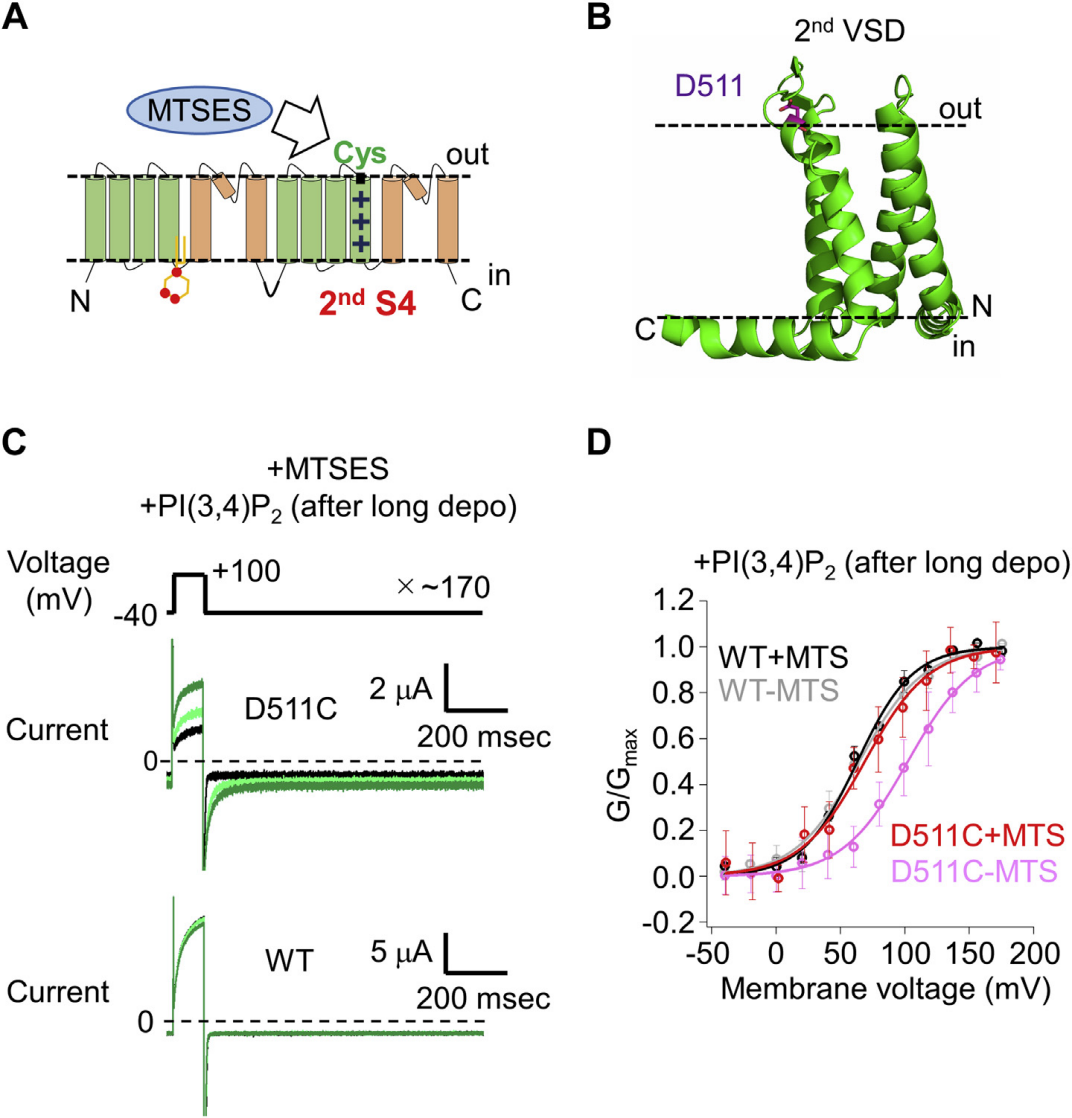
**Characterization of D511C-TPC3 for Cys-accessibility analysis.***A*, a schematic presentation of the two repeats-type structure of TPC3 and the Cys-accessibility analysis using MTSES to detect structural change of the second S4 of TPC3. VSD and PD are colored in *green* and *brown*, respectively. The arginine residues in the second VSD are depicted as +. PI(3,4)P_2_ is also depicted around the binding site. *B*, a homology model of the second VSD in the XtTPC3 structure. It is based on the structure of mouse TPC1 (PDBID: 6C9A) (19). D511, which was mutated to Cys in this study is presented as *sticks*. *C*, representative TPC3 current traces recorded in the presence of 0.5 mM MTSES. These experiments were performed in the presence of PI(3,4)P_2_. The current traces at the beginning (*black*), in the middle (*light green*), and at the end of the experiment (*green*) are shown to represent the MTSES-dependent current change. The *upper panel* shows current traces of D511C-TPC3 and the *lower panel* shows those of TPC3 (WT). *D*, the G-V relationships of WT or D511C-TPC3 after incubation without or with 0.5 mM MTSES in the presence of PI(3,4)P_2_. The color codes are as follows. *Gray*: WT without MTSES, *black*: WT with MTSES, *pink*: D511C-TPC3 without MTSES, and *red*: D511C-TPC3 with MTSES. The V_1/2_ values and slope factors are as follows. WT without MTSES (V_1/2_: 64.1 ± 4.1 mV and slope factor: 25.5 ± 3.7 mV (n = 3)), WT with MTSES (V_1/2_: 63.1 ± 3.1 mV and slope factor: 21.3 ± 1.4 mV (n = 3)), D511C-TPC3 without MTSES (V_1/2_: 111.6 ± 24.3 mV and slope factor: 26.9 ± 12.1 mV (n = 8)), and D511C-TPC3 with MTSES (V_1/2_: 69.2 ± 12.8 mV and slope factor: 25.9 ± 10.0 mV (n = 5)). The results of the statistical analyses of the V_1/2_ values are as follows (One-way ANOVA with Tukey’s test): WT without MTSES *versus* WT with MTSES: *p* = 1.000, WT without MTSES *versus* D511C-TPC3 without MTSES: *p* = 0.012, and D511C-TPC3 without MTSES *versus* D511C-TPC3 with MTSES: *p* = 0.008. *p* < 0.05 was considered statistically significant. G-V relationship, conductance-voltage relationship; MTSES, 2-sulfonatoethyl methanethiosulfonate sodium salt; PD, pore domain; S4, the fourth helix; TPC, two-pore channel; V_1/2_, membrane voltage for half maximum activation; VSD, voltage sensor domain; XtTPC3, *Xenopus tropicalis* TPC3.

The movement of the second S4 was evaluated in *Xenopus* oocytes heterologous expression system by measuring the modification rate by 2-sulfonatoethyl methanethiosulfonate sodium salt (MTSES), which covalently modifies the cysteine residue (Cys) introduced into the second S4. Upon application of MTSES to TPC3 with a Cys residue in the second S4, the MTSES-modification of the introduced Cys is expected to affect the voltage dependence and current of TPC3 (Fig. 2*A*). The rate of this modification depends on the degree of exposure of the introduced Cys to the bath solution. Therefore, possible change in the movement of the second S4 due to PI(3,4)P_2_ binding could be detected as the difference in the rate of the modification in the absence and presence of PI(3,4)P_2_.

First, we searched for the optimal position of the Cys-introduction in TPC3. D511C mutation in the extracellular side of the second S4 shifted strongly the membrane voltage for half maximum activation (V_1/2_) of TPC3 to the depolarized direction (Fig. 2, *B* and *D*, WT V_1/2_: 64.1 ± 4.1 mV (n = 3) and D511C V_1/2_: 111.6 ± 24.3 mV (n = 8)). We note that immediately before the analysis of these conductance-voltage (G-V) relationships, ‘long depolarization’ stimulus (+50 mV, >10 s) was applied to increase the concentration of PI(3,4)P_2_ through the endogenous voltage-dependent PI(3,4)P_2_ production system of *Xenopus* oocytes.

Covalent binding of negatively charged MTSES at the neutral Cys511 is expected to mimic the original negative charge of D511 and restore the WT property, resulting in the significant change of TPC3 current. Firstly, the effect of MTSES was examined in the presence of PI(3,4)P_2_. The application of MTSES increased the current of D511C-TPC3, whereas no obvious change was observed in WT TPC3 (Fig. 2*C*). As expected, the G-V relationship of D511C-TPC3 in the presence of PI(3,4)P_2_ was shifted to the hyperpolarized direction by MTSES-modification, which explains the current increase by MTSES at constant voltage, in contrast to the lack of the shift for WT TPC3 (Fig. 2*D*, WT without MTSES V_1/2_: 64.1 ± 4.1 mV (n = 3) and WT with 0.5 mM MTSES V_1/2_: 63.1 ± 3.1 mV (n = 3); D511C without MTSES V_1/2_: 111.6 ± 24.3 mV (n = 8) and D511C with 0.5 mM MTSES V_1/2_: 69.2 ± 12.8 mV (n = 5)).

**Figure 3:**
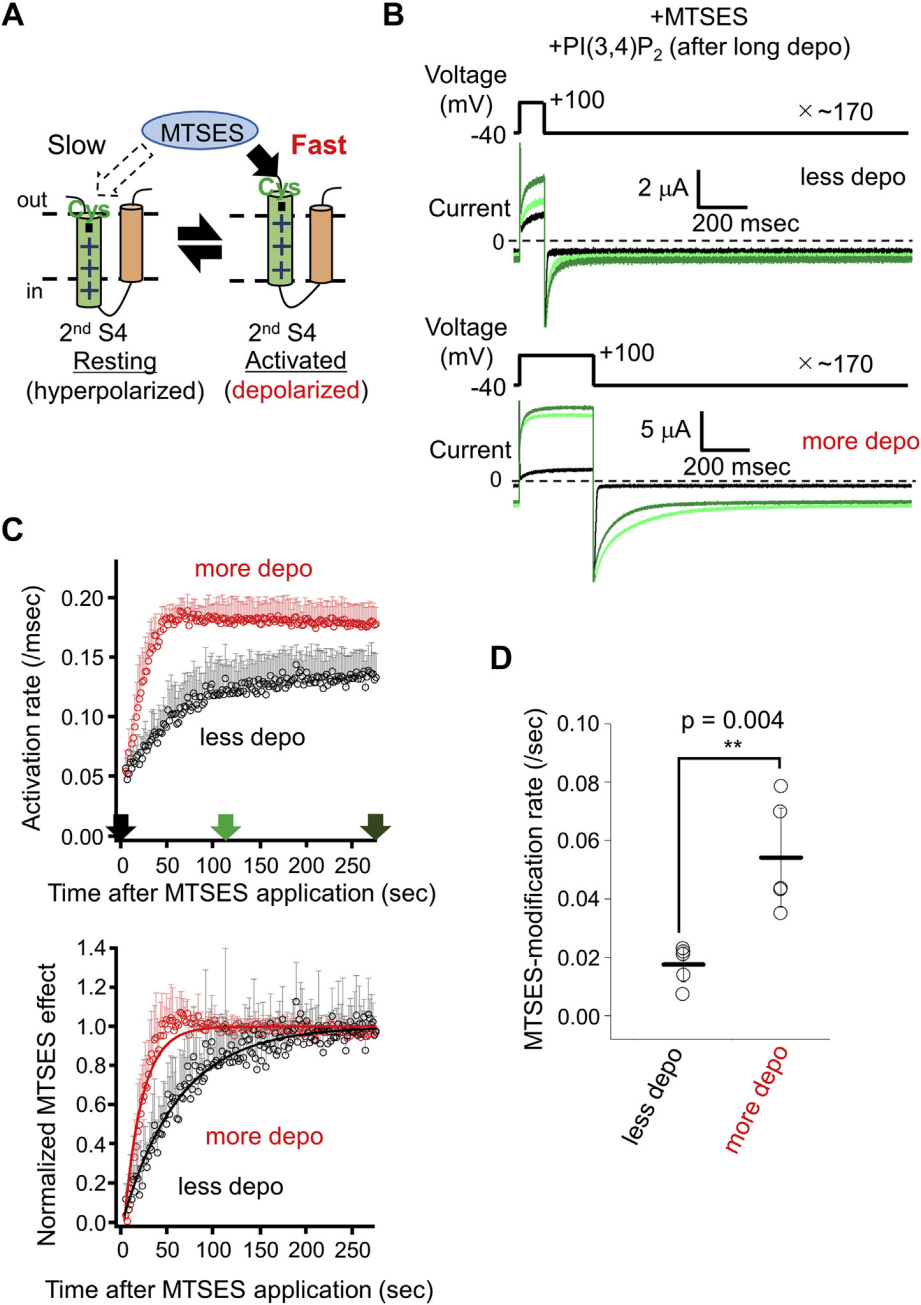
**Modification of D511C by MTSES is state-dependent.***A*, a schematic view of the possible voltage dependence of the MTSES-modification rate due to the movement of the second S4. *B*, representative TPC3 current traces recorded in the presence of 0.5 mM MTSES using two protocols with different lengths of depolarization (depicted as ‘less depo’ and ‘more depo’). These experiments were performed in the presence of PI(3,4)P_2_. The traces are shown in the same manner as Figure 2*C*. *C*, *upper panel*, time-lapse change of the activation rates. The activation rates of TPC3 were obtained, as described in Experimental procedures. The data in *black* are the results by the ‘less depo’ protocol, and the ones in *red* are those by the ‘more depo’ protocol. The *arrows* indicate the points in which the traces in Figure 3*B* were recorded (indicated by the same colors). *Lower panel*, time-lapse change of the normalized MTSES effect. The color codes are the same as those in the *upper panel*. The *solid lines* show the results of single exponential fit. The data points around 50 s for ‘more depo’ protocol slightly deviate from the fitting curve due to the contamination of gradual decrease in the activation rates observed in the late phase of the recording. This is presumably because of the slight decrease in the PI(3,4)P_2_ concentration caused by extensively repeated depolarizations. The error bars only show the *upper side* of the S.D. values for clarity (n = 5). For both plots, the data at the very beginning of the MTSES application are omitted because of their current instability. *D*, statistical comparison of the MTSES-modification rates obtained using two protocols. ‘Less depo’ protocol: 0.018 ± 0.006/s (n = 5) and ‘more depo’ protocol: 0.054 ± 0.017/s (n =5) (*p* = 0.004 by unpaired *t* test. ∗∗ means a statistically significant difference of *p* < 0.01). MTSES, 2-Sulfonatoethyl methanethiosulfonate sodium salt; S4, the fourth helix; TPC, two-pore channel.

To investigate the voltage dependence of the modification rate (Fig. 3*A*), two protocols were used in the presence of PI(3,4)P_2_ (Fig. 3*B*). One is “less depolarization protocol” in which 0.1 s depolarization every 1.6 s is repeated in the presence of MTSES. The other is “more depolarization protocol” with 0.3 s depolarization every 1.6 s. To evaluate the MTSES-modification rate, the TPC3 current was fitted by a double exponential function, and the two activation rates for each pulse were calculated. The activation rate of the slow component was plotted as a function of time from the start of MTSES application (Fig. 3*C*). After normalization, the MTSES-modification rates were calculated by single exponential fitting (Fig. 3, *C* and *D*). In the more depolarization protocol, the MTSES-modification rate was larger than that in the less depolarization protocol (Fig. 3*D*). This result indicates that the movement of the second S4 upon depolarization exposed D511C to external bath solution (Fig. 3*A*). Altogether, D511C-TPC3 and MTSES can be used to detect the structural change of the second S4; faster modification rate indicates the higher occupancy of the second S4 in the depolarized conformation.

### Analysis of the effect of PI(3,4)P_2_ binding on the second S4

**Figure 4:**
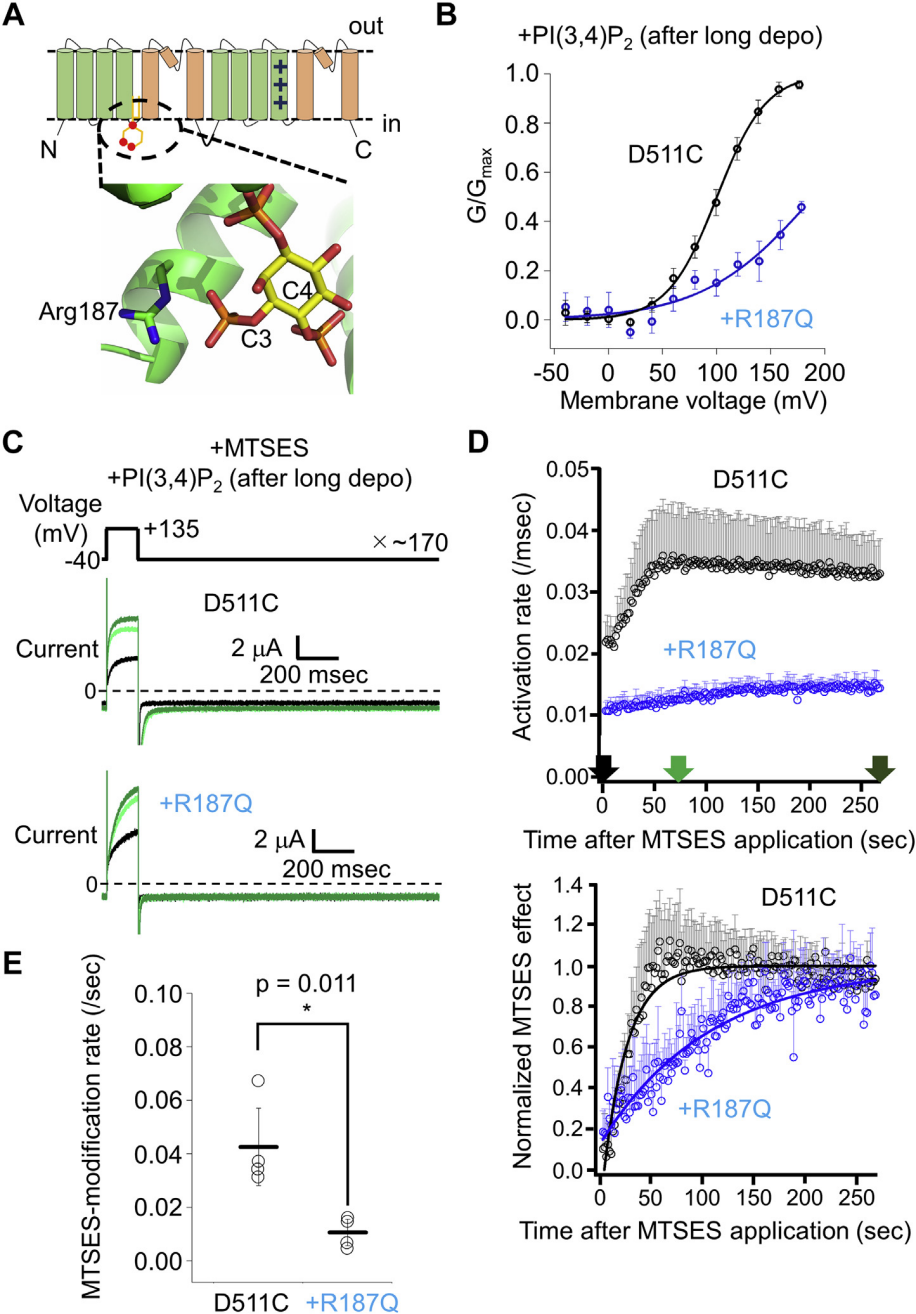
**Analysis of the effect of R187Q mutation in D511C-TPC3.***A*, the model of the binding mode of PI(3,4)P_2_ in the first repeat in XtTPC3. This model is based on the structure of mouse TPC1 (PDBID: 6C9A) (19). Arg187, which is mutated to Gln in this experiment and the modeled PI(3,4)P_2_ are depicted as *sticks*. The positions of the carbon atoms bound to phosphate groups are indicated (C3 and C4). *B*, the G-V relationships of D511C-TPC3 (*black*) and R187Q&D511C-TPC3 (*blue*) without modification by MTSES in the presence of PI(3,4)P_2_. The V_1/2_ values and slope factors are as follows. D511C-TPC3 (V_1/2_: 100.5 ± 4.8 mV and slope factor: 22.9 ± 2.8 mV (n = 10)) and R187Q&D511C-TPC3 (V_1/2_: 180.0 ± 50.1 mV and slope factor: 46.1 ± 20.9 mV (n = 8)). The difference of the V_1/2_ values was statistically significant (*p* < 0.001 by unpaired *t* test). *C*, representative TPC3 current traces recorded in the presence of 0.5 mM MTSES. These experiments were performed in the presence of PI(3,4)P_2_. The traces are shown in the same manner as Figure 2*C*. The *upper panel* shows current traces of D511C-TPC3 (D511C), and the *lower panel* shows those of R187Q&D511C-TPC3 (+R187Q). *D*, *upper* and *lower panels* are shown in the same manner as Figure 3*C*. *Upper panel*, time-lapse change of the activation rates. The activation rates of TPC3 were obtained, as described in Experimental procedures. The data in *black* are the results of D511C-TPC3 and the ones in *blue* are those of R187Q&D511C-TPC3. *Lower panel*, time-lapse change of the normalized MTSES effect. The color codes are the same as those in the *upper panel*. The data points around 50 s for D511C-TPC3 slightly deviate from the fitting curve due to the contamination of gradual decrease in the activation rates observed in the late phase of the recording, similar to the result shown in Figure 3*C*. The error bars only show the *upper side* of the S.D. values for clarity (n = 4). For both plots, the data at the very beginning of the MTSES application are omitted because of their current instability. *E*, statistical comparison of the MTSES-modification rates of two constructs. D511C-TPC3: 0.043 ± 0.014/s (n = 4); R187Q&D511C-TPC3: 0.011 ± 0.005/s (n = 4) (*p* = 0.011 by unpaired *t* test. ∗ means a statistically significant difference of *p* < 0.05). G-V relationship, conductance-voltage relationship; MTSES, 2-Sulfonatoethyl methanethiosulfonate sodium salt; TPC, two-pore channel; V_1/2_, membrane voltage for half maximum activation; XtTPC3, Xenopus tropicalis TPC3.

Next, we examined the effect of PI(3,4)P_2_ binding on the MTSES-modification rate of D511C-TPC3. For this experiment, it is necessary to control the level of PI(3,4)P_2_. Long depolarization activates endogenous voltage-sensitive phosphatase (VSP) in *Xenopus* oocyte which produces PI(3,4)P_2_. Therefore, first, we tried to analyze the difference of the MTSES-modification rates with or without a prepulse of long depolarization (in the presence and the absence of PI(3,4)P_2_, respectively). However, under the condition without long depolarization, we observed gradual current increase irrespective of the application of MTSES. This is possibly because of the activation of endogenous VSP, which caused an increase in PI(3,4)P_2_ concentration during the experiment by repeated short depolarizations. This PI(3,4)P_2_-induced current increase obscured the detection of MTSES-induced current increase. To solve this problem, an alternative approach was taken using R187Q&D511C-TPC3 mutant. We previously reported that the positive charge at position 187 in the first S4–S5 linker is essential for PI(3,4)P_2_ binding (25) (Fig. 4*A*). The MTSES-modification rates were compared between D511C-TPC3 and R187Q&D511C-TPC3 in the presence of PI(3,4)P_2_. The protocol used here includes depolarizing pulse of +135 mV, which is expected to effectively discriminate between the activation states of D511C-TPC3 and R187Q&D511C-TPC3 based on their G-V relationships in the presence of PI(3,4)P_2_ (Fig. 4*B*, D511C-TPC3 V_1/2_: 100.5 ± 4.8 mV (n = 10) and R187Q&D511C-TPC3 V_1/2_: 180.0 ± 50.1 mV (n = 8)). Both constructs showed MTSES-induced increases in the current amplitude (Fig. 4*C*) and the activation rate (Fig. 4*D*). When the MTSES-modification rates were calculated based on the current amplitude, it was hard to discriminate the modification rate of D511C-TPC3 from that of R187Q&D511C-TPC3, due to the instability of the current amplitude during experiment. When calculated based on the activation rate, the MTSES-modification rate of D511C-TPC3 was significantly larger than that of R187Q&D511C-TPC3 (Fig. 4*E*). This result indicates that the second S4 of D511C-TPC3 is more frequently exposed to the extracellular solution by +135 mV depolarization than that of R187Q&D511C-TPC3, which could be attributed to the reduced PI(3,4)P_2_ binding caused by R187Q mutation.

**Figure 5:**
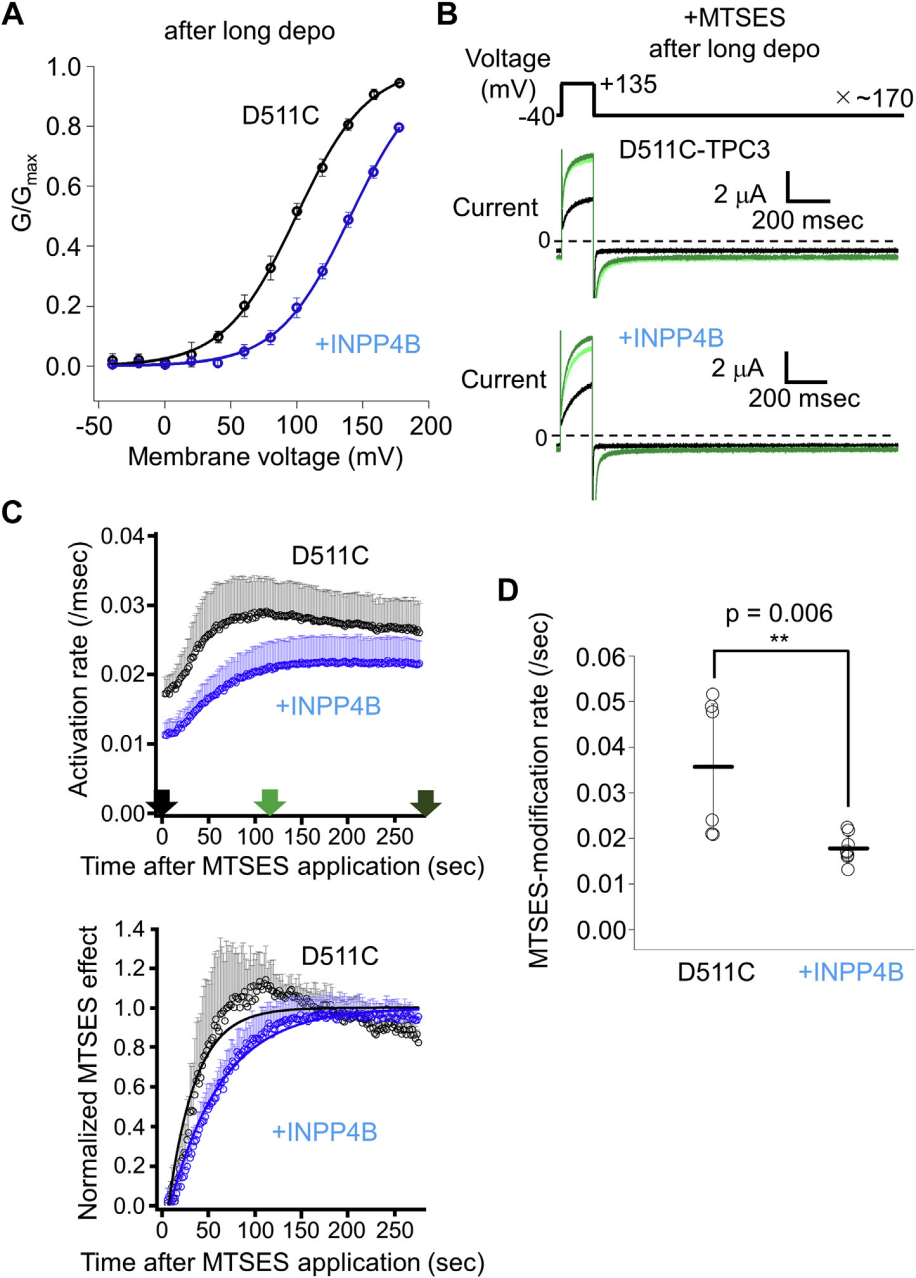
**Analysis of the effect of INPP4B coexpression with D511C-TPC3.***A*, the G-V relationships of D511C-TPC3 only and coexpressed with INPP4B without modification by MTSES after long depolarization. The color codes are as follows: *black*: D511C-TPC3 and *blue*: D511C-TPC3+INPP4B. The V_1/2_ values and slope factors are as follows: D511C-TPC3 (V_1/2_: 99.5 ± 3.4 mV and slope factor: 27.4 ± 2.9 mV (n = 6)) and D511C-TPC3+INPP4B (V_1/2_: 140.4 ± 3.5 mV and slope factor: 27.4 ± 2.4 mV (n = 6)). The difference of the V_1/2_ values was statistically significant (*p* < 0.001 by unpaired *t* test). *B*, representative TPC3 current traces recorded in the presence of 0.5 mM MTSES with or without coexpression of INPP4B. These experiments were performed after long depolarization. The traces are shown in the same manner as Figure 2*C*. *C*, *upper panel*, time-lapse change of the activation rates. The color codes are the same as those in Figure 5*A*. *Lower panel*, time-lapse change of the normalized MTSES effect (Experimental procedures). The color codes are the same as those in the *upper panel*. The *upper* and the *lower panels* are shown in the same manner as Figure 3*C*. The data points around 75 s for D511C-TPC3 deviate from the fitting curve due to the contamination of gradual decrease in the activation rates observed in the late phase of the recording, similar to the result shown in Figure 3*C*. The error bars only show the *upper side* of the S.D. values for clarity (n = 6 for D511C-TPC3, n = 8 for D511C-TPC3+INPP4B). For both plots, the data at the very beginning of the MTSES application are omitted because of their current instability. *D*, statistical comparison of the MTSES-modification rates between D511C-TPC3 only and D511C-TPC3 coexpressed with INPP4B. D511C-TPC3: 0.036 ± 0.014/s (n = 6) and D511C-TPC3+INPP4B: 0.018 ± 0.003/s (n = 8) (*p* = 0.006 by unpaired *t* test. ∗∗ means a statistically significant difference of *p* < 0.01). G-V relationship, conductance-voltage relationship; INPP4B, inositol polyphosphate 4-phosphatase type II; MTSES, 2-Sulfonatoethyl methanethiosulfonate sodium salt; TPC, two-pore channel; V_1/2_, membrane voltage for half maximum activation.

To further prove the effect of PI(3,4)P_2_ binding on the MTSES-modification of D511C-TPC3, we took another approach using a PI(3,4)P_2_-degrading enzyme, human inositol polyphosphate 4-phosphatase type II (INPP4B) (28, 29, 30). INPP4B is a phosphatase that specifically dephosphorylates PI(3,4)P_2_. Therefore, heterologous expression of INPP4B is expected to maintain low concentration of PI(3,4)P_2_ in oocytes even after long depolarization. D511C-TPC3 was coexpressed with INPP4B. G-V relationships analyzed after long depolarization showed that coexpression of INPP4B caused a positive shift of the G-V relationship (Fig. 5*A*, D511C-TPC3 V_1/2_: 99.5 ± 3.4 mV (n = 6) and D511C-TPC3+INPP4B V_1/2_: 140.4 ± 3.5 mV (n = 6)). The MTSES-modification rate of D511C-TPC3+INPP4B was smaller than that of D511C-TPC3 (Fig. 5, *B*–*D*). These results indicate that depletion of PI(3,4)P_2_ rendered the extracellular side of the second S4 less exposed during the experiment. This is consistent with the result about R187Q mutant described above, in which deficiency of PI(3,4)P_2_ binding decelerated the MTSES-modification (Fig. 4).

### Analysis of the structural rearrangement using voltage clamp fluorometry

To detect structural changes of membrane proteins, VCF is a useful method, in which specific amino acid residues are labeled by fluorescent molecule one at a time. The change in the fluorescence intensity (F change) reports local structural change (31). Based on the canonical model of the S4 movement, the extracellular and intracellular termini of the second S4 are expected to undergo significant changes in their chemical environment upon activation, for example, from the lipid bilayer phase to the water phase or vice versa, indicating that these regions can be good labeling sites for VCF analysis using environment-sensitive fluorophores. Therefore, the structural rearrangement of the second S4 of TPC3 was investigated using constructs fluorescently labeled at the residues in these regions of the second S4.

**Figure 6:**
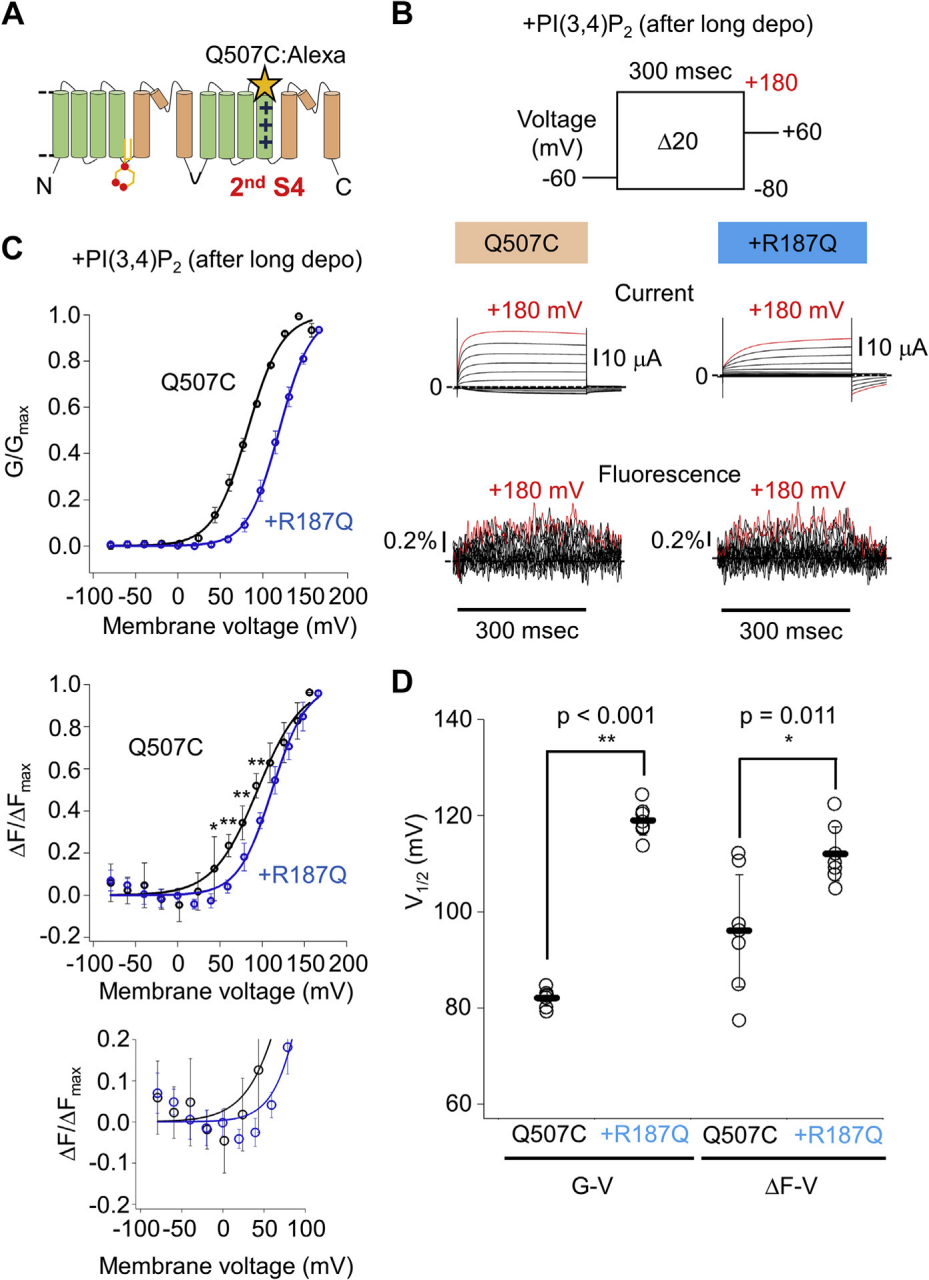
**VCF analysis using TPC3 with fluorescent label in the extracellular side of the second S4 and comparison with the PI(3,4)P**_**2**_**-binding deficient mutant.***A*, a schematic view of the position of Q507C. *B*, representative results showing TPC3 current and fluorescence traces of Q507C-TPC3 (*left*) and R187Q&Q507C-TPC3 (*right*). These experiments were performed in the presence of PI(3,4)P_2_. The traces shown in *red* are the current and fluorescent traces at +180 mV, respectively. *C*, the G-V (*upper panel*) and ΔF-V (*middle panel*) relationships of Q507C-TPC3 (*black*) and R187Q&Q507C-TPC3 (*blue*) in the presence of PI(3,4)P_2_. The V_1/2_ values and slope factors are as follows: Q507C-TPC3 (V_1/2_ of G-V: 82.1 ± 1.7 mV and slope factor of G-V: 20.4 ± 2.0 mV; V_1/2_ of ΔF-V: 96.1 ± 11.7 mV and slope factor of ΔF-V: 26.9 ± 5.2 mV (n = 7)) and R187Q&Q507C-TPC3 (V_1/2_ of G-V: 119.0 ± 3.1 mV and slope factor of G-V: 18.4 ± 1.4 mV; V_1/2_ of ΔF-V: 112.0 ± 5.6 mV and slope factor of ΔF-V: 20.1 ± 1.8 mV (n = 7)). In the ΔF-V relationships, the results of statistical comparison of the ΔF/ΔF_max_ values at each membrane voltage are indicated when they showed statistical significance. *p* values are shown in Table S1 (unpaired *t* test). The *lower panel* shows the expanded view of the *middle panel* (ΔF-V), focusing on the first phase. A slight increase in the ΔF/ΔF_max_ value at −80 mV suggests a possibility that holding potential (−60 mV) is within the range of the membrane voltage of the first phase. *D*, statistical comparison of the V_1/2_ values described above (G-V: *p* < 0.001, ΔF-V: *p* = 0.011 by unpaired *t* test. ∗ and ∗∗ mean statistically significant differences of *p* < 0.05 and *p* < 0.01, respectively). ΔF-V relationship, F change-voltage relationship; G-V relationship, conductance-voltage relationship; S4, the fourth helix; TPC, two-pore channel; V_1/2_, membrane voltage for half maximum activation; VCF, voltage clamp fluorometry.

Covalent labeling of Cys at the targeted position using a fluorescent dye conjugated with maleimide is a canonical method for VCF analysis (31, 32, 33, 34, 35, 36, 37). After screening of the optimal labeling site (Fig. S1), Q507 was found to be an appropriate position as a labeling site in the extracellular side of the second S4 (Fig. 6*A*, Q507C-TPC3). After incubation of the oocytes expressing Q507C-TPC3 with Alexa Fluor 488 C5 Maleimide, TPC3 current and F change were simultaneously recorded in the presence of PI(3,4)P_2_ (Fig. 6*B*). The amplitude of the F change evoked by +180 mV depolarizing pulse was 0.39 ± 0.18% (n = 7). This small amplitude of the F change made it necessary to repeat the test pulses 20 times and to average the obtained traces to achieve sufficiently high-signal/noise ratio.

### Analysis of the effect of PI(3,4)P_2_ binding on Q507C-TPC3 by VCF technique

Similar analysis of TPC3 current and F change in the absence of PI(3,4)P_2_ was difficult because the repeated depolarization protocol to increase the signal/noise ratio activates the endogenous VSP in the oocyte. Therefore, the PI(3,4)P_2_ binding deficient mutant (R187Q&Q507C-TPC3) was analyzed to mimic the condition in the absence of PI(3,4)P_2_ after long depolarization (Fig. 6*B*). The G-V and F change-voltage (ΔF-V) relationships of Q507C-TPC3 and R187Q&Q507C-TPC3 were obtained in the presence of PI(3,4)P_2_ (Fig. 6*C*, Q507C-TPC3 V_1/2_ of G-V: 82.1 ± 1.7 mV, V_1/2_ of ΔF-V: 96.1 ± 11.7 mV (n = 7) and R187Q&Q507C-TPC3 V_1/2_ of G-V: 119.0 ± 3.1 mV, V_1/2_ of ΔF-V: 112.0 ± 5.6 mV (n = 7)). The statistical comparison between Q507C-TPC3 and R187Q&Q507C-TPC3 showed the significant difference of both the V_1/2_ values of G-V and ΔF-V (Fig. 6*D*). This suggests that the PI(3,4)P_2_ binding shifts the voltage dependence of the movement of the second S4 toward the hyperpolarized direction, resulting in the potentiation of the voltage dependent-activation of TPC3.

In contrast to the well-fitted sigmoidal curve in the G-V relationship, the ΔF-V relationship of Q507C-TPC3 apparently showed a biphasic character, composed of the first small decrease and the following main increase in the ΔF/ΔF_max_ (Fig. 6*C*). Fig. S2 shows representative F changes of Q507C-TPC3 evoked by multiple depolarizing or hyperpolarizing pulses from −60 mV. The fluorescence intensity slightly decreased upon the weak depolarization to 0 mV, whereas it clearly increased upon the strong depolarization to +180 mV, consistent with the biphasic character in the ΔF-V relationship. Unfortunately, we could not analyze the first phase and the second phase individually because of the overlap of the two phases in the ΔF-V relationship. The first phase emerged in the more hyperpolarized voltage range compared with the gate-opening shown in the G-V relationship (Fig. 6*C*). In contrast, the second phase emerged in a voltage range similar to that of the gate-opening (Fig. 6*C*).

This biphasic character was also observed in the ΔF-V relationship of R187Q&Q507C-TPC3. ΔF/ΔFmax values at each membrane voltage were compared (Fig. 6*C* and Table S1). ΔF/ΔFmax values at +40 ∼ +100 mV, in which the second phase is dominant, showed statistical difference, whereas those at −80 ∼ +20 mV, in which the first phase is dominant, did not. This result suggests that PI(3,4)P_2_ binding affects at least the second phase of the movement of the second S4. Owing to the small F changes in the first phase, we could not conclude clearly whether they are affected by PI(3,4)P_2_ binding or not (Fig. 6*C*, lower panel and Fig. S2).

**Figure 7:**
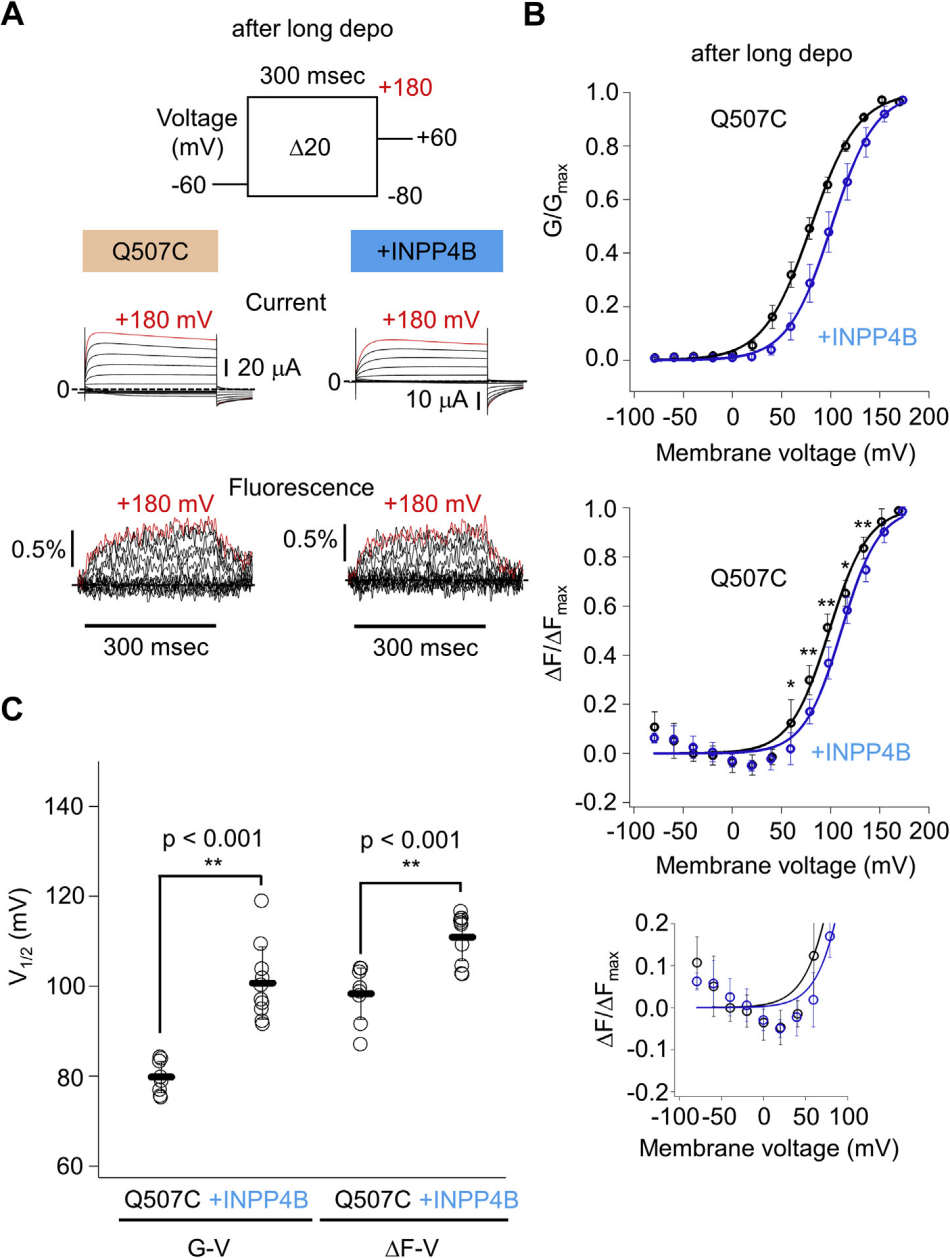
**VCF analysis of Q507C-TPC3 coexpressed with INPP4B.***A*, representative results showing the TPC3 current and fluorescence traces of Q507C-TPC3 (*left*) and Q507C-TPC3 coexpressed with INPP4B (*right*). These experiments were performed after long depolarization. The traces shown in *red* are the current and fluorescent traces at +180 mV, respectively. *B*, the G-V (*upper panel*) and ΔF-V (*middle panel*) relationships of Q507C-TPC3 (*black*) and Q507C-TPC3 coexpressed with INPP4B (*blue*) after long depolarization. The V_1/2_ values and slope factors are as follows: Q507C-TPC3 (V_1/2_ of G-V: 79.8 ± 3.5 mV and slope factor of G-V: 20.4 ± 2.2 mV; V_1/2_ of ΔF-V: 98.3 ± 5.7 mV and slope factor of ΔF-V: 19.5 ± 1.4 mV (n = 8)) and Q507C-TPC3 + INPP4B (V_1/2_ of G-V: 100.8 ± 8.0 mV and slope factor of G-V: 22.0 ± 1.1 mV; V_1/2_ of ΔF-V: 111.0 ± 5.2 mV and slope factor of ΔF-V: 19.1 ± 2.3 mV (n = 10)). In the ΔF-V relationships, the results of statistical comparison of the ΔF/ΔF_max_ values at each membrane voltage are indicated when they showed statistical significance. *p* values are shown in Table S2 (unpaired *t* test). The *lower panel* shows the expanded view of the *middle panel* (ΔF-V), focusing on the first phase. A slight increase in the ΔF/ΔF_max_ value at −80 mV suggests a possibility that holding potential (−60 mV) is within the range of the membrane voltage of the first phase. *C*, statistical comparison of the V_1/2_ values described above (G-V: *p* < 0.001, ΔF-V: *p* < 0.001 by unpaired *t* test. ∗∗ means a statistically significant difference of *p* < 0.01). ΔF-V relationship, F change-voltage relationship; G-V relationship, conductance-voltage relationship; INPP4B, inositol polyphosphate 4-phosphatase type II; TPC, two-pore channel; V_1/2_, membrane voltage for half maximum activation.

In addition to the analysis of the PI(3,4)P_2_-binding deficient mutant described above, the effect of the coexpression of the PI(3,4)P_2_-degrading enzyme (INPP4B) on G-V and ΔF-V relationships of Q507C-TPC3 was also analyzed (Fig. 7 and Table S2). The coexpression of INPP4B caused statistically significant shifts of both the V_1/2_ values of G-V and ΔF-V compared with the case without coexpression (Fig. 7), although the F changes in the first phase in the ΔF-V were again not large enough to analyze any possible shift in the first phase caused by the INPP4B coexpression (Fig. 7*B*, lower panel). These results, together with those obtained using R187Q mutation, suggest that the binding of PI(3,4)P_2_ potentiates the voltage dependence of the movement of the second S4 at least in the second phase, which results in enhanced voltage-dependent activation.

### Measurement of the gating charges of TPC3 in mammalian cells

**Figure 8:**
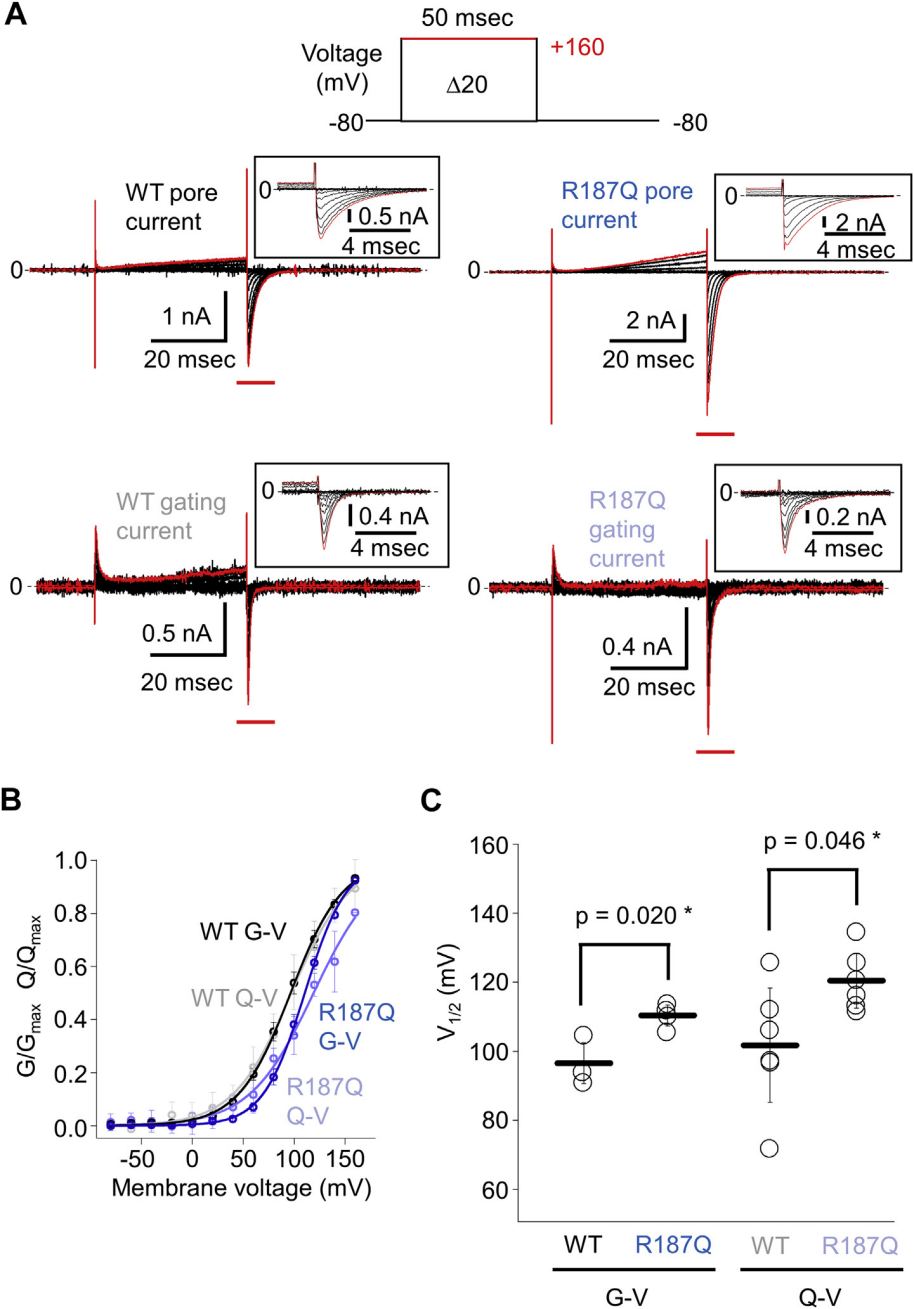
**Gating charge recordings of WT TPC3 and R187Q-TPC3.***A*, representative results showing the ‘pore current’ (*upper panels*) and ‘gating current’ (*lower panels*) of WT TPC3 (*left panels*) and R187Q-TPC3 (*right panels*). These traces were recorded from the transfected HEK293T cells by whole cell-patch clamp technique. The protocol for voltage step pulses is shown on the *top*. The traces shown in *red* are the current traces at +160 mV. The pipette solution did not contain Na^+^, which is the major permeating ion of TPC3. For the recordings of ‘pore current’, bath solution contained Na^+^ to detect TPC3 tail current attributed to the permeation through TPC3 channel pore. For the recordings of ‘gating current’, bath solution did not contain Na^+^ to detect gating current attributed to the S4 movement without contamination by pore current upon the repolarization to −80 mV from the step pulses. The traces in the time range marked by *red bars* are expanded in the *insets*. These experiments were performed with an assumption of the presence of remaining PI(3,4)P_2_ according to a previous report (38). *B*, G-V and Q-V relationships of WT TPC3 and R187Q-TPC3 (R187Q). The color codes are as follows: *Black*: WT G-V, *blue*: R187Q G-V, *gray*: WT Q-V, and *pale blue*: R187Q Q-V. The V_1/2_ values and slope factors are as follows: WT TPC3 (V_1/2_ of G-V: 96.7 ± 5.8 mV and slope factor of G-V: 25.6 ± 1.2 mV (n = 3); V_1/2_ of Q-V: 101.7 ± 16.5 mV and slope factor of Q-V: 29.6 ± 4.8 mV (n = 6)) and R187Q-TPC3 (V_1/2_ of G-V: 110.3 ± 3.0 mV and slope factor of G-V: 20.4 ± 1.4 mV (n = 4); V_1/2_ of Q-V: 120.3 ± 7.9 mV and slope factor of Q-V: 31.9 ± 2.0 mV (n = 6)). *C*, statistical comparison of the V_1/2_ values described above (G-V: *p* = 0.020, Q-V: *p* = 0.046 by unpaired *t* test. ∗ means a statistically significant difference of *p* < 0.05). G-V relationship, conductance-voltage relationship; Q-V relationship, gating charge-voltage relationship; S4, the fourth helix; TPC, two-pore channel; V_1/2_, membrane voltage for half maximum activation.

VCF analysis revealed that the ΔF-V relationship of Q507C-TPC3 shows two phases (Figs. 6*C* and 7*B*). To assign explicitly each component to the gating charge movement, we recorded the gating currents that reflect the transfer of charged particles in S4 helix in TPC3. Owing to the technical difficulty in measuring the gating currents in *Xenopus* oocytes, we used patch-clamp technique in the HEK293T cells expressing TPC3. In the mammalian cells, PI(3,4)P_2_ is considered to be contained in plasma membrane to some extent (38). Therefore, we assumed a continuous presence of sufficient level of PI(3,4)P_2_ for TPC3. The gating currents of TPC3 were successfully recorded under the solution containing no permeant cations in both bath and pipette solutions. They appeared as transient and fast currents, distinguishable from the normal-pore currents observed in the presence of Na^+^ in bath solution (Fig. 8*A*). The gating charge-voltage (Q-V) relationship was determined by integrating the amplitude of gating currents generated by repolarizations to -80 mV from step pulses (see Experimental procedures). In HEK293T cells, the G-V and Q-V were shifted to the depolarized direction than those recorded in oocytes (Figs. 2*D* and 8*B*), possibly because of the difference in the cells used, or to the washout of the components maintaining channel activity by the replacement of the intracellular contents with the pipette solution. The value of V_1/2_ of Q-V (101.7 mV ± 16.5 mV) was almost identical to that of G-V (96.7 mV ± 5.8 mV). These overlapped Q-V and G-V are reminiscent of the ΔF-V and G-V in VCF measurement of Q507C-TPC3 (Fig. 6, *C* and *D*, 7, *B* and *C* and 8*B*). However, no additional component at lower voltage was observed in the measurement of gating charges, in contrast to the observation of the first phase in the ΔF-V of Q507C-TPC3. These results suggest that the second phase in the ΔF-V reflects the movement of gating charges, and that the first phase represents the movement of the second S4 involving no or undetectable charge transfer.

In addition, to investigate whether or not the gating charges are affected by PI(3,4)P_2_ binding to the first repeat in TPC3, we performed the same measurements using R187Q-TPC3 mutant. Both Q-V and G-V relationships of R187Q-TPC3 were shifted toward the depolarized direction compared with those of WT (Fig. 8, *B* and *C*). These results clearly indicate that PI(3,4)P_2_ binding potentiates the transfer of the gating charges of TPC3.

### Fluorescent labeling of the intracellular side of the second S4 using a fluorescent unnatural amino acid

Next, the intracellular side of the second S4 was examined as another target for labeling. Because the Cys-based labeling method is not applicable to the intracellular side, a fluorescent unnatural amino acid, Anap (3-(6-acetylnaphthalen-2-ylamino)-2-aminopropanoic acid), was used. Anap can label TPC3 at any position because it is incorporated during protein translation (39).

**Figure 9:**
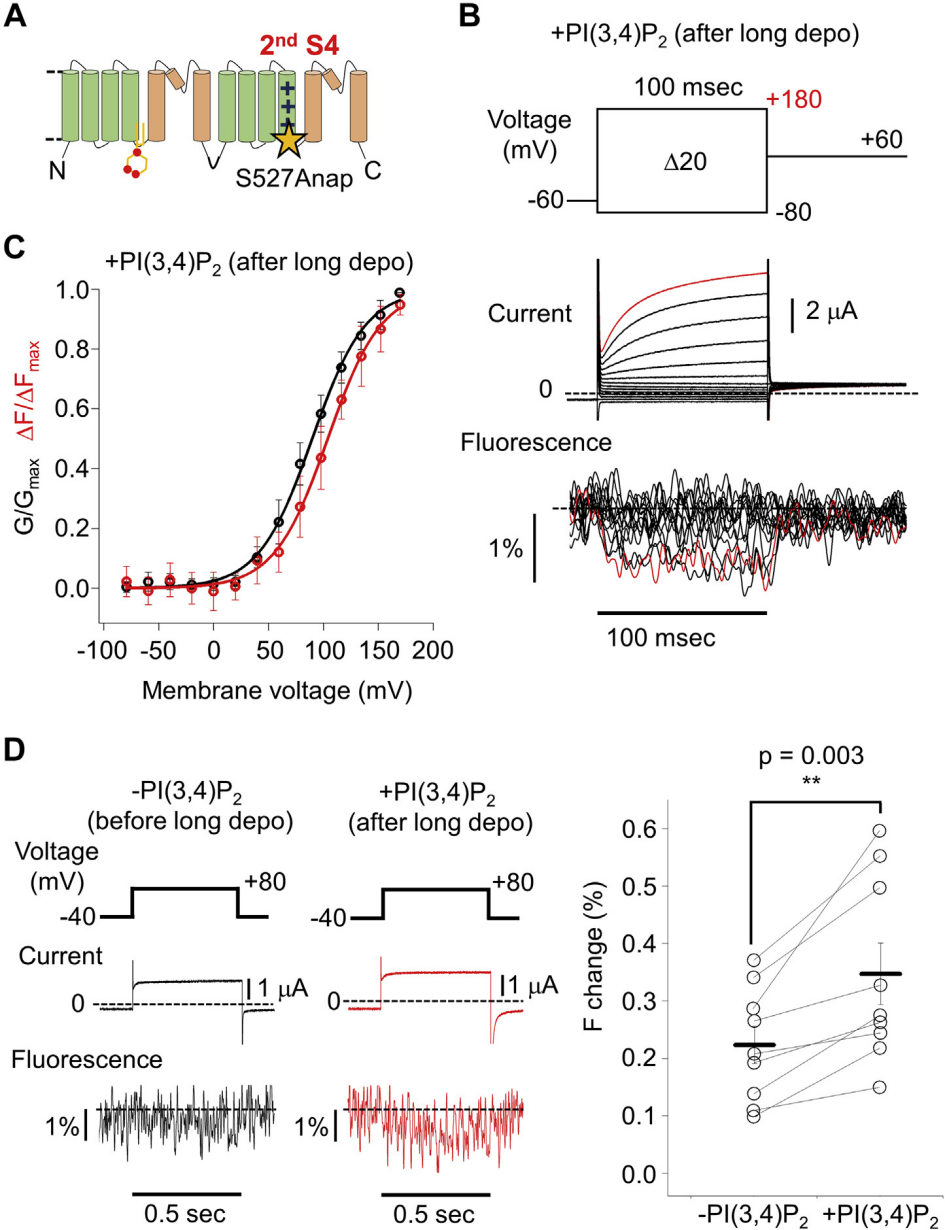
**VCF analysis using TPC3 with fluorescent label in the intracellular side of the second S4.***A*, a schematic view of the position of S527Anap. *B*, a representative result showing TPC3 current and fluorescence traces of S527Anap-TPC3. These experiments were performed in the presence of PI(3,4)P_2_. The traces shown in *red* are the current and fluorescent traces at +180 mV, respectively. *C*, the G-V (*black*) and ΔF-V (*red*) relationships of S527Anap-TPC3 in the presence of PI(3,4)P_2_. The V_1/2_ values and slope factors are as follows: V_1/2_ of G-V: 89.7 ± 7.6 mV and slope factor of G-V: 24.0 ± 1.1 mV; V_1/2_ of ΔF-V: 106.0 ± 9.9 mV and slope factor of ΔF-V: 24.9 ± 7.7 mV (n = 12). *D*, *left* and *middle*, representative results showing S527Anap-TPC3 current and fluorescence traces before (*black*) and after (*red*) long depolarization. The long depolarization is expected to increase PI(3,4)P_2_ concentration in the oocyte. These traces were recorded from the same oocyte. *Right*, statistical comparison of the F changes of S527Anap-TPC3 before and after long depolarization. The averages of the detected F changes are as follows: before long depolarization: 0.16 ± 0.09%; after long depolarization: 0.29 ± 0.15% (n = 9) (*p* = 0.003 by paired *t* test. ∗∗ means a statistically significant difference of *p* < 0.01). ΔF-V relationship, F change-voltage relationship; Anap, 3-(6-acetylnaphthalen-2-ylamino)-2-aminopropanoic acid; F change, change of fluorescence intensity; G-V relationship, conductance-voltage relationship; S4, the fourth helix; TPC3, two-pore channel; V_1/2_, membrane voltage for half maximum activation; VCF, voltage clamp fluorometry.

By screening multiple amino acid residues in the intracellular side as labeling sites (Fig. S3), we observed that S527Anap-TPC3 showed a clear depolarization-evoked decrease in the fluorescence intensity (Fig. 9, *A* and *B*). The direction of the F change for S527Anap-TPC3 (decrease upon depolarization) is different from that for Q507C-TPC3 (mainly increase upon depolarization). Multiple factors such as the type of the fluorophores and/or the environmental change upon voltage change around the introduced location could influence the direction of the F changes, resulting in the difference between Q507C (Alexa dye) and S527Anap. The averaged amplitude of the F change of S527Anap-TPC3 evoked by +180 mV depolarizing pulse was 0.92 ± 0.50% (n = 12). The G-V and ΔF-V relationships were obtained in the presence of PI(3,4)P_2_ (Fig. 9*C*, V_1/2_ of G-V: 89.7 ± 7.6 mV and V_1/2_ of ΔF-V: 106.0 ± 9.9 mV (n = 12)). We note that any biphasic character in the ΔF-V relationship was not clearly observed for S527Anap-TPC3 (Fig. 9*C*). S527Anap-TPC3 might report only the movement of the second S4 corresponding to the second phase in Q507C-TPC3, given their voltage dependences (see Figs. 6*C*, 7*B* and 9*C*). Therefore, the first phase occurring at significantly hyperpolarized membrane voltage to the gating was uniquely detected by Q507C-TPC3, with a label on the extracellular side. This result might indicate that it represents any extracellular conformational rearrangement that is not immediately linked to the channel gating.

Because of the limited expression of R187Q + S527Anap-TPC3 and S527Anap-TPC3+INPP4B, it was not possible to analyze G-V and ΔF-V relationships of S527Anap-TPC3 mimicking PI(3,4)P_2_-unbound state. Instead, we compared the F changes of S527Anap-TPC3 evoked by short single depolarizing pulses, that is, in the absence and in the presence of PI(3,4)P_2_. The F change of S527Anap-TPC3 was sufficiently large in the presence of PI(3,4)P_2_, enabling us to obtain reliable F change by a single depolarizing pulse (*i.e.*, without repeat of the test pulses to increase the signal/noise ratio). Because a single short depolarizing pulse does not activate endogenous VSP sufficiently, it was also suitable for the recording in the absence of PI(3,4)P_2_ (Fig. 9*D*). Using the short pulses, the current and fluorescence traces were recorded both in the absence and in the presence of PI(3,4)P_2_ from the same oocytes by applying a long depolarization in between the short pulses (Fig. 9*D*). When analyzed with the short depolarizing pulse at +80 mV, the recorded TPC3 current was slightly potentiated in the presence of PI(3,4)P_2_ compared with that in the absence of PI(3,4)P_2_ (Fig. 9*D*), presumably because of the shift of the G-V relationship. Furthermore, although there was almost no F change in the absence of PI(3,4)P_2_, a significantly larger voltage-dependent F change was detected at the same voltage in the presence of PI(3,4)P_2_ (0.16 ± 0.09% in the absence of PI(3,4)P_2_ *versus* 0.29 ± 0.15% in the presence of PI(3,4)P_2_ (n = 9), Fig. 9*D*). These VCF results indicate that the binding of PI(3,4)P_2_ potentiates the voltage-dependent structural change not only in the extracellular side (Figs. 6 and 7) but also in the intracellular side of the second S4.

**Figure 10:**
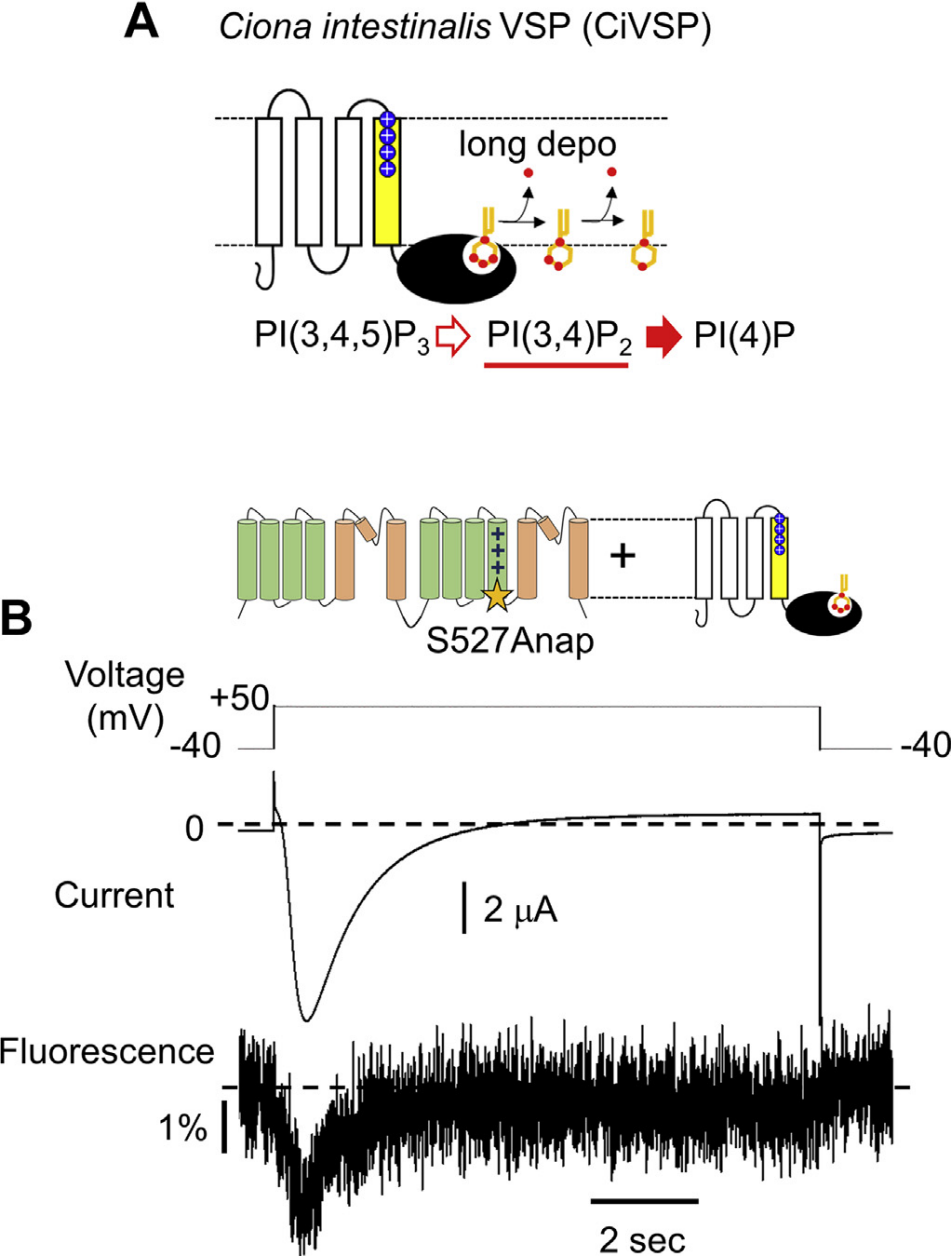
**Coexpression of CiVSP with S527Anap-TPC3 evokes a rapid and biphasic F change.***A*, a schematic explanation of the enzymatic activity of CiVSP. *B*, a representative result of the coexpression experiment of S527Anap-TPC3 with CiVSP, in which CiVSP activated by the long depolarization transiently produced and then degraded PI(3,4)P_2_. Anap, 3-(6-acetylnaphthalen-2-ylamino)-2-aminopropanoic acid; CiVSP, Ciona intestinalis VSP; F change, change of fluorescence intensity; TPC, two-pore channel; VSP, voltage sensitive phosphatase.

Next, to further demonstrate the relationship between PI(3,4)P_2_ binding to the first repeat and the movement of the second S4, exogenous VSP was coexpressed with S527Anap-TPC3 to dramatically change the concentration of PI(3,4)P_2_ in a shorter time (Fig. 10*A*). VSP from *Ciona intestinalis* (CiVSP) is known to dephosphorylate phosphatidylinositol (3,4,5) trisphosphate in a stepwise manner on the plasma membrane. Depolarization stimulus to CiVSP results in the production of PI(3,4)P_2_ from phosphatidylinositol (3,4,5) trisphosphate, followed by phosphatidylinositol (4) phosphate production from PI(3,4)P_2_ (40, 41). Therefore, in CiVSP-expressing oocytes, the concentration of PI(3,4)P_2_ transiently increases and then decreases during the sustained depolarization, which is expected to affect the current and fluorescence of S527Anap-TPC3. VCF analysis showed that the inward current of S527Anap-TPC3 transiently increased and then decreased upon the activation of the coexpressed CiVSP (Fig. 10*B*). The fluorescence intensity of S527Anap-TPC3 also showed a biphasic change similar to that of the current. The time scales of these changes in both current and fluorescence intensity were in a similar range to that of the change in PI(3,4)P_2_ concentration evoked by CiVSP activation, reported in previous studies using fluorescence-based sensor specific for PI(3,4)P_2_ (25, 40, 41). This result indicates that the amplitude of the current and the fluorescence intensity derived from S527Anap-TPC3 were well correlated with the PI(3,4)P_2_ concentration, showing that TPC3 current changes rapidly in response to changes in PI(3,4)P_2_ concentration, which is attributed to the potentiation of the structural changes of the second S4 by PI(3,4)P_2_.

## Discussion

**Figure 11:**
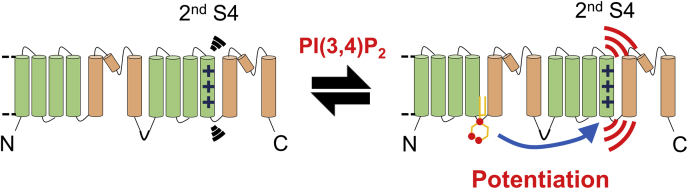
**The effect of PI(3,4)P**_**2**_**binding on the second S4.** A schematic explanation of the potentiation of the structural change of the second S4 by PI(3,4)P_2_ binding to the first repeat revealed by this study. This ‘global modulation’ in TPC3 is the molecular mechanism underlying the potentiation of the voltage dependence of TPC3 gating by PI(3,4)P_2_. S4, the fourth helix; TPC3, two-pore channel.

TPC3 is regulated by both membrane voltage and PI (25). In this study, we investigated how these two stimuli are dynamically integrated in TPC3. The effect of the PI(3,4)P_2_ binding to the first repeat on the voltage-dependent movement of the second S4 voltage sensor was analyzed by three kinds of methods: Cys-accessibility analysis using MTSES, VCF, and measurement of gating charges. These analyses revealed that PI(3,4)P_2_ binding remotely controls the voltage-dependent structural rearrangement of the second S4 (Fig. 11).

### Significance of the inter-repeat ‘global modulation’

In this research, we raised and examined two possibilities as the mechanism of the PI(3,4)P_2_-dependent potentiation in TPC3: ‘local modulation’ around the pore gate and ‘global modulation’ involving the second S4 (Fig. 1). The obtained data here showed that the effect of PI(3,4)P_2_ binding to the first repeat is not confined to the pore gate but reaches the second S4 that is away from the PI(3,4)P_2_-binding site (Figs. 1 and 11), showing that the ‘global modulation’ does occur. The ‘global modulation’ requires the effect of the PI(3,4)P_2_ binding to be transmitted from the intracellular side of the first repeat (PI(3,4)P_2_-binding site) to the S4 in the second VSD (Fig. 1) (25). We speculate that the signal of PI(3,4)P_2_ binding to the first repeat is transmitted to the second repeat in the same subunit through the inter-repeat interaction in between the first S6 and the adjacent second S6 of the same subunit, which was shown to be necessary for the PI(3,4)P_2_-dependent potentiation (25). However, there remains a possibility that the signal is also transmitted to the second S4 in the other subunit through the interaction between four S6s, which requires further investigation. In any case, we speculate that this ‘transmission’ across the almost whole molecule of TPC3 could make the effect of the PI(3,4)P_2_ binding on the voltage-dependent gating of TPC3 more drastic, than the possible case of the ‘local modulation’ limited to the pore gate, resulting in the significant shift of the voltage dependence. Furthermore, as discussed later, this ‘global modulation’ might be the basis for the integration of multiple signals by TPCs, which is a general feature of this protein family.

### Voltage-dependent structural change of the second S4 of TPC3 and the effect of PI(3,4)P_2_

The Q-V relationship showed only one phase (Fig. 8*B*). Based on the range of the voltage at which this phase occurs, it was shown that the second phase in the ΔF-V relationship of Q507C corresponds to the major movement of the second S4 that transfers gating charges during depolarization-induced activation (Figs. 6*C*, 7*B* and 8*B*). Unlike Q507C, S527Anap-TPC3 showed ΔF-V relationship with only a single phase, but its V_1/2_ value is again in close range to that of Q-V (Fig. 9*C*). This coincidence indicates that they are likely to reflect the same dynamic structural change of the second S4 with the movement of the gating charges (42), which occurs across the whole helix from the extracellular to the intracellular sides. In addition, our detailed analyses showed that PI(3,4)P_2_ consistently potentiates the Q-V, ΔF-Vs, and G-Vs, as well as the MTSES accessibility to the extracellular region of the second S4. The results show that the PI(3,4)P_2_ binding modulates the dynamic structural rearrangement of the whole second S4 helix, rather than that of a limited region.

The ΔF-V and Q-V that represent the movement of gating charges generally precedes the G-V in voltage-gated channels, because there is a cooperative step with inter-subunit interaction for the final opening of the gate after the gating charges have been transferred in multiple S4s (27, 37, 43, 44, 45, 46, 47, 48). However, in TPC3, the V_1/2_ values of ΔF-Vs and the Q-V are consistently close to those of G-Vs (Figs. 6*D*, 7*C* and 8*C*). These overlaps indicate two unique features of TPC3. First, the second S4 movement may be more tightly associated with gate-opening in TPC3 than in canonical voltage-gated channels, enabling TPC3 to be gated without any cooperative final step. Second, the movement of only one of the two second S4s in TPC3 channel could be enough to open the gate. Otherwise, after one S4 has moved, the channel could not open till another S4 moves, causing an additional step and a gap between G-V and Q-V (27, 37, 43, 44, 45, 46, 47, 48). Related to this hypothesis, it is reported that, in KCNQ1 channel, the movement of one of the four S4s is enough to partially open the gate, demonstrated by mutational experiment to lock S4 movement (49). TPC3 might have an analogous mechanism to that of KCNQ1.

The features discussed above enables us to describe the possible gating mechanism of the second S4, in which a drastic movement of the whole second S4, driven by the transfer of gating charges immediately leads to the gate opening that does not require the final state transition. The binding of PI(3,4)P_2_ to the first repeat robustly modulates this dynamic movement of the distally located second S4 through the inter-repeat interaction. An interesting, but unresolved point regarding the second S4 movement is the first phase in the ΔF-V of Q507C-TPC3 at less depolarized membrane voltages. Our Q-V analysis of TPC3 showed that there is no or undetectable gating current in the voltage range of the first phase in the ΔF-V. Therefore, the first phase is not likely to involve a large charge transfer across the membrane. Given the unique features of TPC3 that the major second S4 movement (the second phase) could be strongly associated with the opening of the gate, it is possible that the movement of the second S4 has an ‘unique first phase’ occurring in the extracellular side with undetectable charge transfer, preparing for the second phase. Further investigation is needed to examine this hypothesis.

### Comparison with the S4 movement in the Nav channel

TPCs are evolutionally close to four repeats-type of Nav channels (50). The voltage-dependent movements of the four S4s in Nav have been evaluated through VCF analysis (36, 51). Nav showed inter-repeat coupling of S4s that was demonstrated *via* the analysis showing that the mutational perturbation of one of the S4s also affects the movement of other S4s in different repeats (36). Combining with our present results showing significant coupling between the first repeat and the second repeat in TPC3, tight inter-repeat coupling might be a common feature among multi-repeats type of voltage-gated cation channels. This coupling was proposed to enable the fast activation kinetics of Nav and complex coupling between activation and inactivation (36). In TPC3, the coupling of the second S4 to other repeat is thought to be harnessed to achieve the regulation by PI(3,4)P_2_ binding to the first repeat. Therefore, our present study revealed an example of using the tight coupling of S4 to integrate multiple physiological signals and could have a broad implication for complex regulatory mechanisms of multi-repeats type of ion channels.

### The possible generality of the ‘global modulation’ in TPC family

The ‘global modulation’ in TPC3 by PI(3,4)P_2_ indicates that TPC3 molecule is coupled across the whole molecule to be regulated by both membrane voltage and PI(3,4)P_2_ (Fig. 1). This coupling within TPC molecule is also observed in the structure of TPC1 from *Arabidopsis thaliana* (AtTPC1) (52). In AtTPC1, Ca^2+^ binding to the intracellular domain potentiates the voltage-dependent activation (53). Single particle structure analysis by cryo-EM and crystallographic structure analysis showed that the intracellular Ca^2+^ binding reciprocally affects the structural rearrangement of the extracellular region of the second VSD in AtTPC1 (52). This coupling crossing over the membrane region enables AtTPC1 to integrate the signals of Ca^2+^ binding and depolarization. Because integration of various signals is a shared function of TPC family (18, 22, 25, 53, 54), the ‘global modulation’ and/or tight coupling across the whole molecule might be a general feature for TPC family members. In our study, optical experiment (VCF) significantly contributed to reveal the coupling of TPC3 molecule. To our knowledge, this is the first optical study of the structural changes of TPC family protein. Therefore, this study could serve as a basis for future analysis of the dynamic structural rearrangements of TPCs, which would provide further mechanistic insights into the physiological regulation of TPCs.

## Experimental procedures

### Ethical approval

All animal experiments were approved by the Animal Care Committee of the National Institutes of Natural Sciences and performed in accordance with the guidelines.

### Molecular biology

The cDNAs of TPC3 from XtTPC3 (XP_002940387) and HsINPP4B (NP_001095139) were subcloned into pGEMHE vector, as described previously (25). The CiVSP cDNA was provided by Prof. Okamura (Osaka University). For gating charge recordings using HEK293T cell, the cDNA of XtTPC3 was subcloned into pCXN2 expression vector. Site-directed mutagenesis was accomplished by a PCR-based method using PrimeSTAR Max DNA Polymerase and the In-Fusion HD Cloning Kit (Takara Bio), following the manufacturer’s protocol. The plasmid sequences were confirmed by DNA sequencing. The complimentary RNAs (cRNAs) were transcribed using a mMESSAGE mMACHINE T7 kit (Ambion; Life Technologies) from the linearized cDNA by Nhe1 restriction enzyme (Toyobo). The tRNA-synthetase/Anap-CUA encoding plasmid (pAnap) was obtained from Scripps Research Institute. The salt form of fluorescent unnatural amino acid, Anap, (Futurechem) was dissolved in water and stocked at −20 °C.

### Injection into *Xenopus* oocytes

The females of *Xenopus laevis* for oocytes isolation were purchased from Hamamatsu Seibutsu Kyouzai. The oocytes were collected by surgical operation from frogs anesthetized by 0.15% tricaine. After the final collection, the frogs were humanely sacrificed by decapitation. The isolated oocytes were treated with 2 mg/ml collagenase (Sigma-Aldrich) for 6.5 h and incubated overnight or longer at 17 °C in frog Ringer’s solution containing (in mM) 88 NaCl, 1 KCl, 2.4 NaHCO_3_, 0.3 Ca(NO_3_)_2_, 0.41 CaCl_2_, 0.82MgSO_4_, and 15 Hepes, pH 7.6, with 0.1% penicillin–streptomycin (Sigma-Aldrich). The oocytes were then injected with 50 nl of cRNA solution and kept at 17 °C in frog Ringer’s solution. Depending on the expression level of the constructs, the amount of injected cRNAs of XtTPC3 per oocyte ranged 2.5 ∼ 25 ng for Cys-accessibility analysis and 12.5 ∼ 25 ng for VCF. In VCF using Anap, first, 0.5 ng pAnap was injected into the nucleus of oocyte. On the next day, 50 nl of a solution containing 25 pmol of Anap and 12.5 ∼ 25 ng cRNA were injected into cytoplasm. In the case of coexpression experiments with CiVSP, the mixture of 25 ng of XtTPC3 cRNA and 2.5 ng of CiVSP cRNA were injected. In the case of coexpression experiments with HsINPP4B for Cys accessibility analysis, the mixture of 5 ng of XtTPC3 cRNA and 1.25 ng of HsINPP4B cRNA were injected. In the case of coexpression experiments with HsINPP4B for VCF analysis, the mixture of 20 ng of XtTPC3 cRNA and 5 ng of HsINPP4B cRNA were injected. The currents were recorded 2 to 6 days after injection, depending on the current amplitude required.

### Two-electrode voltage clamp recording of oocytes

The currents were recorded under two-electrode voltage clamps using an OC-725C amplifier (Warner Instruments) and pClamp10.4 software (Molecular Devices). The data from the amplifier were digitized at 10 kHz through Digidata1440A (Molecular Devices). The resistance of the microelectrodes was 0.2 to 1.0 MΩ when filled with a solution of 3 M K-acetate and 10 mM KCl. The standard recording bath solution was ND-96, which contains (in mM) 96 NaCl, 2 KCl, 1.8 CaCl_2_, 1 MgCl_2_, and 5 Hepes, pH 7.4. Unless noted otherwise, the holding potential was at −40 mV. In Cys-accessibility analysis, after obtaining stable current by repeated depolarization pulses, 0.5 mM MTSES (Biotium) in ND-96 was applied to the bath by perfusion. The recordings were performed at room temperature.

### Manipulation of PI(3,4)P_2_ level in *Xenopus* oocytes

To control PI(3,4)P_2_ concentration, VSP which is endogenously expressed in *Xenopus* oocyte was used (41), except for the experiment using heterologous CiVSP. Long depolarization (*e.g.*, +50 mV, ∼10 s) activates endogenous VSP and produces PI(3,4)P_2_ at the plasma membrane. Therefore in this article, ‘the absence of PI(3,4)P_2_’ or ‘without PI(3,4)P_2_’ means ‘before long depolarization’, whereas ‘the presence of PI(3,4)P_2_’ or ‘with PI(3,4)P_2_’ means ‘after long depolarization’. In the analyses of the G-V and ΔF-V relationships of TPC3, 100 ∼ 500 ms of +100 mV depolarization was applied before every step pulse to confirm that the TPC3 current was stable, indicating the stable PI(3,4)P_2_ binding during the experiments.

### Analysis of the MTSES-modification rate

The current amplitude of D511C-TPC3 (a construct used in Cys-accessibility analysis) was not stable enough before MTSES application depending on oocyte batches, but the activation kinetics of TPC3 was rather stable. Thus, the kinetics was better for the analysis of the MTSES-modification. Each of the TPC3 current traces obtained by the repeated depolarization pulses was fitted by a double exponential function, and the activation rate was calculated. For the analysis in Figure 3, *C* and *D*, the rate constant of the slow component was used for the analysis, because it was more stable during recording than that of the fast component (the contribution of the slow component was ∼40%). For the analysis in Figures 4, *D* and *E* and 5, *C* and *D*, the rate constant of the fast component was used for its higher stability (the contribution of the fast component was 60∼80%). MTSES-induced changes in the TPC3 activation rate were obtained by subtracting the activation rate just before MTSES application (rate(t = 0)) from the activation rates recorded at each time point (rate(t)). The obtained values were then normalized to the calculated maximum increase in the activation rate (rate(t = ∞)-rate(t = 0)), to show clearly the difference in the modification rates between different conditions (*e.g.*, protocols and constructs). After plotting the normalized value as a function of time, MTSES-modification rate was obtained by fitting the plots using single exponential functions.

### Voltage clamp fluorometry

In the case of VCF using Alexa Fluor 488 C5 Maleimide (Thermo Fisher Scientific), oocytes with the expression of Q507C-TPC3 were labeled with 100 μM Alexa Fluor 488 C5 Maleimide (Thermo Fisher Scientific) in *N*-Methyl _D_-glucamine (NMDG) buffer which contains (in mM) 5 NaCl, 91 NMDG, 2 KCl, 1.8 CaCl_2_, 1 MgCl_2_, and 5 Hepes, pH 7.4 at 4 °C. After that, the oocytes were rinsed and stored in NMDG buffer.

The fluorometric recordings were performed with an upright fluorescence microscope (Olympus BX51WI) equipped with a water immersion objective lens (Olympus XLUMPLFLN 20×/1.00) to collect the emission light from the oocytes. The excitation light from xenon arc lamp (L2194-01, Hamamatsu Photonics) was applied through a band-pass excitation filter (470–490 nm for Alexa488 and 330–360 nm for Anap). To minimize the photobleaching during recording, the intensity of the excitation light was decreased to 1.5 or 6.0% with ND filters (U-25ND6, U-25ND25 Olympus) to decelerate the photobleaching depending on the intensity of the emitted fluorescence. The emission light was passed through the band pass filter: for Alexa488, 510 to 550 nm (U-MNIBA2, Olympus); for Anap, 420 to 460 nm and 460 to 520 nm (Brightline, Semrock) (39, 55). The emission signals were detected by one (for Alexa) or two (for Anap) photomultipliers (H10722-110; Hamamatsu Photonics). The detected fluorescent intensities and TPC3 currents were acquired by the Digidata 1332 (Axon Instruments) and pClamp 10.3 software (Molecular devices) at 20 kHz. To improve the signal to noise ratio, VCF recordings were repeated 20 times for each sample when necessary. The averaged data were used for data presentation and analysis when the recoding was repeated. The excitation light was induced by manually opening the shutter. After opening, the presence of fluorescence was briefly checked, and then oocytes were continuously exposed to the excitation light during the recording, generally resulting in exposure to the excitation light for around 7 min for each oocyte. A slow bleaching achieved by diminished excitation light intensity was adjusted by assuming that the decrease in the fluorescence intensity was linear. Bleaching actually occurred linearly in the time range of our protocol (Fig. S4).

### Patch clamp recording and measurement of the gating charges in mammalian cells

The HEK293T cells were transfected with the plasmid DNAs (μg for 8 × 10^4^ cells) of XtTPC3 (0.8 μg) or its R187Q mutant (0.8 μg) and the plasmid of yellow fluorescent protein as a transfection marker (0.8 μg) using LipofectAMINE 2000 (2 μl, Invitrogen). 24 to 72 h after the transfection, the cells were seeded onto poly-L-Lysine coated cover glasses. After the cells adhered on the glass, the whole cell patch clamp experiments were carried out. The pipette solution contained the following (in mM): 130 NMDG-Cl, 5 MgCl_2_, 10 EGTA, and 10 Hepes, pH 7.3. The composition of the bath solution varies depending on the aim of the experiments. For the recording of gating current, bath solution contained the following (in mM): 140 NMDG-Cl, 2 MgCl_2_, and 10 Hepes, pH 7.4. For the recording of WT TPC3 pore current, it contained the following (in mM): 20 NaCl, 120 NMDG-Cl, 2 MgCl_2_, and 10 Hepes, pH 7.4. For the recording of R187Q-TPC3 pore current, it contained the following (in mM): 140 NaCl, 4 KCl, 1 CaCl_2_, 0.3 MgCl_2_, and 10 Hepes, pH 7.4 to record sufficiently large current. The pipette resistance was 1.5 ∼ 3.0 MΩ, and voltage clamp error due to series resistance was compensated for 70 ∼ 80%. The macroscopic membrane currents were recorded from the cells expressing yellow fluorescent protein using Axopatch 200B amplifiers, Digidata1332A, and pClamp 9 software (Molecular Devices) (56). After establishing the whole cell configuration, the cell was held at −80 mV and step pulses (membrane voltage was increased by +20 mV every pulse, from −80 to +160 mV, pulse length for 50 ms) were applied every 3 s. P/8 protocol (eight sub-pulses opposite to the waveform) was used by pClamp 9 software (Molecular Devices) to eliminate the linear components of membrane currents and capacitative currents from the recorded traces. The data from the amplifier were digitized at 100 kHz.

### Data analysis and statistics

Electrophysiological data were analyzed using Clampfit 10.7 (Molecular Devices) and Igor Pro (WaveMetrics). The tail current amplitude elicited by +60 mV or −80 mV after step pulse was used to obtain G-V relationship. To obtain the change of ΔF-V relationship, the changes of the intensity from the baseline intensity caused by the step pulse were presented as percent of the baseline intensity. To obtain the gating Q-V relationship, the OFF gating current evoked by repolarizations to −80 mV from different membrane voltages were integrated. Although we performed P/8 protocol to subtract the linear component of membrane currents and capacitative currents from the traces, in some traces, the capacitative current could not be completely subtracted. Therefore, integration of the recorded OFF gating current was performed using the current traces in the time period from the time point of settling of the residual capacitative current to the end point of the OFF gating current. The G-V, ΔF-V, and Q-V relationships were calculated by fitting to a sigmoidal function, G, ΔF, or Q = max/(1 + exp((V_1/2_-V)/k)) + base, where G, ΔF, and Q indicate the conductance, the change in the fluorescence intensity, and the gating charge, respectively. V is the membrane voltage and V_1/2_ is the membrane voltage for half maximum activation. k is the slope factor. The normalized G/G_max_, ΔF/ΔF_max_, and Q/Q_max_ were plotted as the functions of membrane voltage. The data are presented as mean ± SD. *t* test or one-way ANOVA with Tukey’s test was used for statistical comparison of two groups or multiple groups, respectively. *p* < 0.05 was considered statistically significant. ∗, ∗∗ denote values of *p* < 0.05, 0.01, respectively. The statistical analyses were performed using Excel (Microsoft) or Igor Pro (WaveMetrics), and the statistical graphs were drawn using Igor Pro (WaveMetrics).

### Three-dimensional structure modeling of *X. tropicalis* TPC3

Homology structure modeling was performed using a web-based environment for protein structure homology modeling SWISS-MODEL (57, 58) based upon sequence alignment of amino acids of XtTPC3 (XP_002940387) and crystal structure of *Mus musculus* TPC1 (PDBID: 6C9A) (19). All the structure figures presented in this study were generated using PyMOL molecular graphics system version 1.7.x (Schrödinger, LLC). The binding model of PI(3,4)P_2_ in the first repeat in TPC3 (Fig. 4*A*) was generated, as described previously (25).

## Data availability

All the data for this study are included within this article.

## Supporting information

This article contains supporting information.

## Conflict of interest

The authors declare that they have no conflicts of interest with the contents of this article.
