# Supplementary material for: Phosphoinositide regulates dynamic movement of the S4 voltage sensor in the second repeat in two-pore channel 3
Source: J Biol Chem. 2021 Nov 18;297(6):101425. doi: 10.1016/j.jbc.2021.101425 (PMC8665364; doi:10.1016/j.jbc.2021.101425)
Supplement: Supplemental Figures S1–S4 and Tables S1, S2 [file mmc1.pdf]

Supporting information for

Phosphoinositide regulates dynamic movement of the S4 voltage sensor in the 2nd repeat in Two-pore channel 3

Ki-ichi Hirazawa, Michihiro Tateyama, Yoshihiro Kubo, Takushi Shimomura

This PDF file includes:

Figure S1-S4

Table S1 and S2

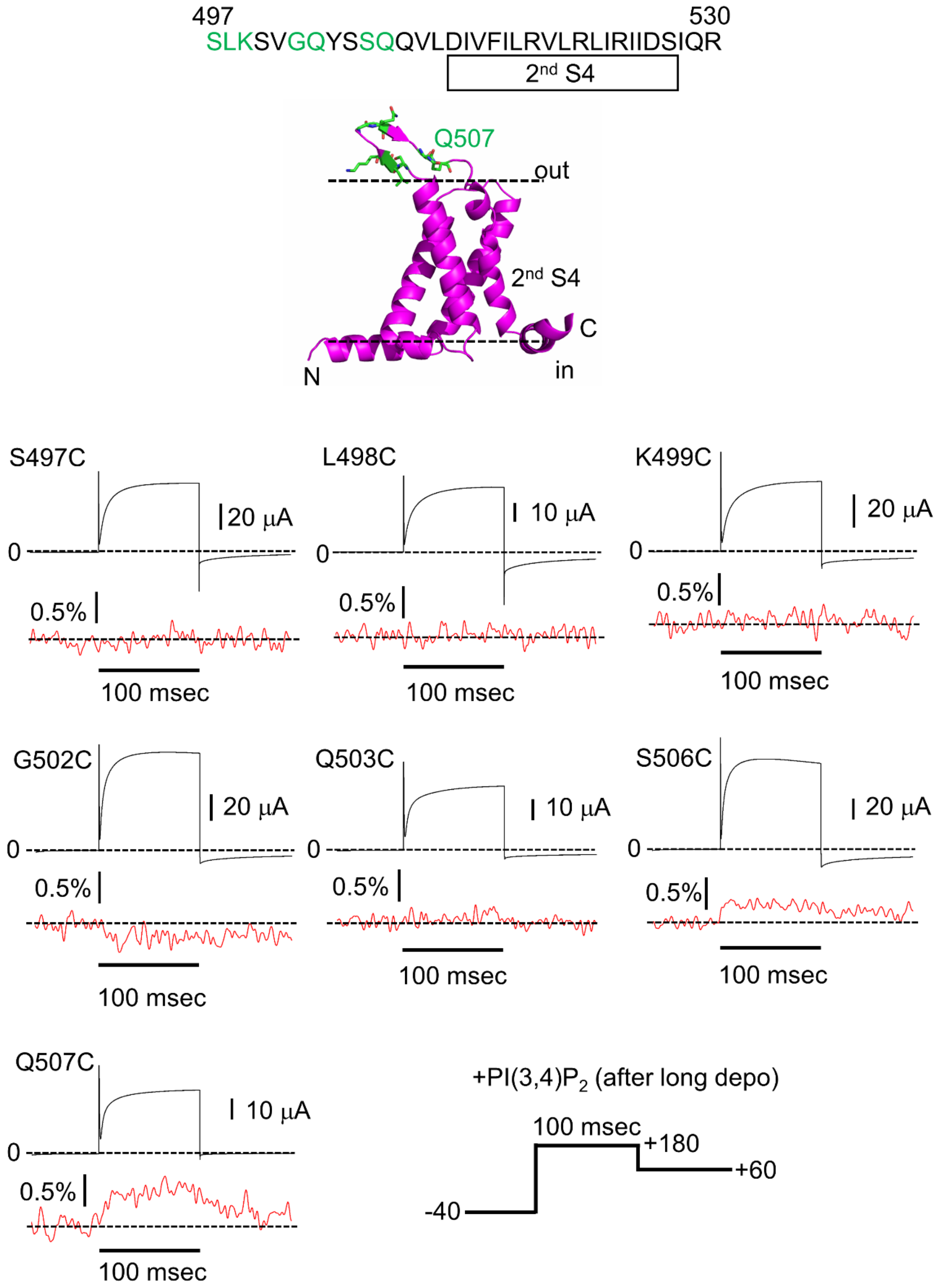
497
530
SLKSVGQYSSQQVLDIVFILRVLRLIRIIDSIQR
2nd S4
Q507
out
2nd S4
C
N
in
S497C
20 μA
0
0.5%
100 msec
L498C
10 μA
0
0.5%
100 msec
K499C
20 μA
0
0.5%
100 msec
G502C
20 μA
0
0.5%
100 msec
Q503C
10 μA
0
0.5%
100 msec
S506C
20 μA
0
0.5%
100 msec
Q507C
10 μA
0
0.5%
100 msec
+PI(3,4)P2 (after long depo)
100 msec
+180
+60
-40

Figure S1. Voltage clamp fluorometry of cysteine mutants of TPC3 labeled at the extracellular side of the 2nd S4

Upper panel: amino acid sequence around the 2nd S4 and homology model of the 2nd VSD structure of *Xenopus tropicalis* TPC3. The residue numbers both at the N-terminal and C-terminal in the displayed amino acid sequence are indicated above the sequence. The residues tested as candidates for Alexa-labeling are colored in green and shown as sticks in the model. Q507, the most successful position for Alexa-VCF, is highlighted in the model. The model is built based on the structure of mouse TPC1 (PDBID: 6C9A). Lower panels: representative results showing TPC3 current traces (black) and fluorescence traces (red) of the 2nd S4 Alexa-constructs. These experiments were performed in the presence of PI(3,4)P_2. The voltage pulse is depicted at the bottom.

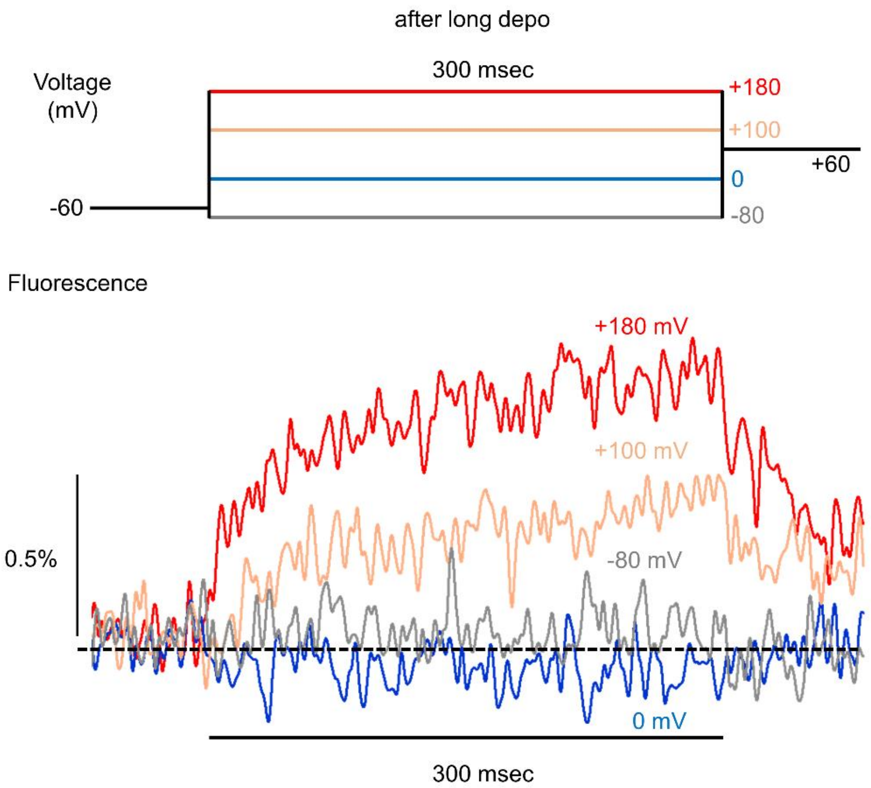

Figure S2. Q507C-TPC3 apparently exhibits biphasic F change

Representative fluorescence traces of Q507C-TPC3. The color codes of the fluorescence traces are as indicated above. The blue fluorescence trace shows slight decrease in the fluorescence intensity upon a weak depolarization to 0 mV in contrast to the clear increase in the fluorescence intensity upon a stronger depolarization to +180 mV (red).

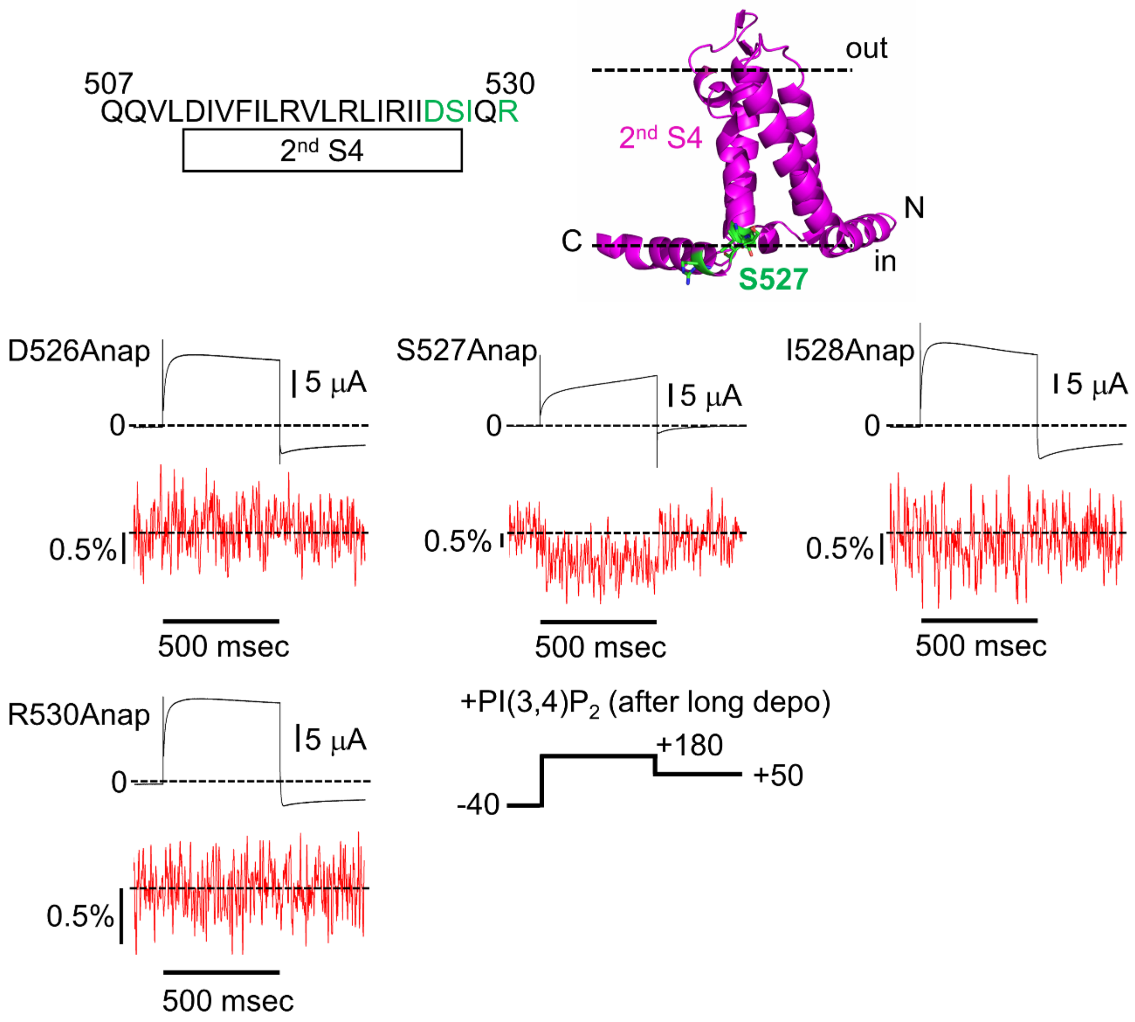

Figure S3. Voltage clamp fluorometry of Anap mutants of TPC3 at the intracellular side of the 2nd S4

Upper panel: amino acid sequence around the 2nd S4 and homology model of the 2nd VSD structure of *Xenopus tropicalis* TPC3. The residue numbers both at the N-terminal and C-terminal in the displayed amino acid sequence are indicated above the sequence. The residues tested as candidates for Anap-labeling are colored green and shown as sticks in the model. S527, the most successful position for Anap-VCF, is highlighted in the model. The model is built in the same manner as that in Fig. S1. Lower panels: representative results showing TPC3 current traces (black) and fluorescence traces (red) of the 2nd S4 Anap-constructs. These experiments were performed in the presence of PI(3,4)P_2. The voltage pulse is depicted at the bottom. The protocol was repeated 3 times for averaging in this figure while 20 times in other figures, resulting in the different appearance.

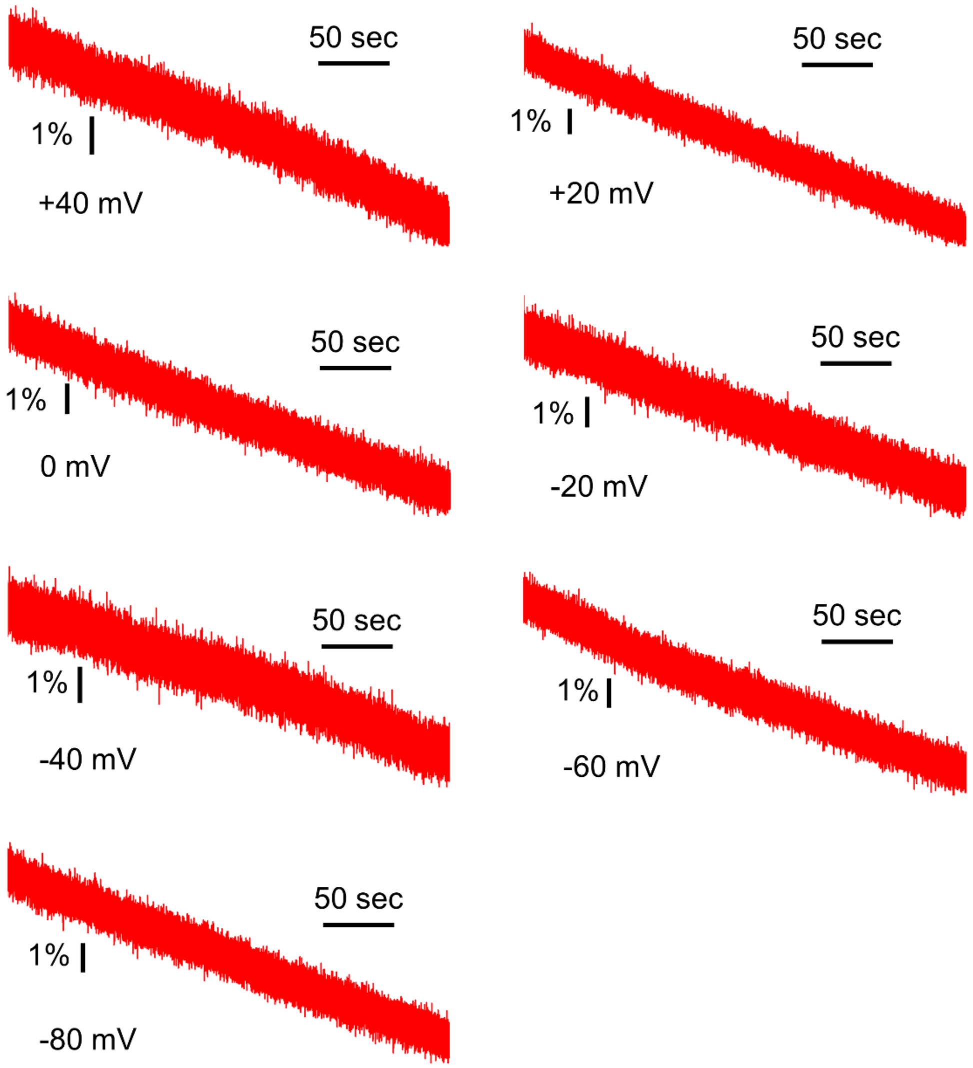

Figure S4. Bleaching of the fluorophore of Alexa Fluor™ 488 C5 Maleimide attached to Q507C-TPC3

Fluorescence traces recorded at each membrane voltage from the oocyte with expression of Q507C-TPC3 labeled by Alexa Fluor™ 488 C5 Maleimide. Each trace was recorded with continuous clamp at the indicated membrane voltage for 300 sec.

Table S1. Results of the statistical comparison of $\Delta F/\Delta F_{max}$ values at each membrane voltage between Q507C-TPC3 and R187Q&Q507C-TPC3

Voltage (mV)	Q507C $\Delta F/\Delta F_{max}$ (±S.D.)	R187Q&Q507C $\Delta F/\Delta F_{max}$ (±S.D.)	p value	significance
-80	0.06±0.09	0.07±0.05	0.798	N.S.
-60	0.02±0.06	0.05±0.03	0.387	N.S.
-40	0.05±0.11	0.01±0.05	0.392	N.S.
-20	-0.02±0.05	-0.01±0.04	0.897	N.S.
0	-0.05±0.08	0.00±0.03	0.228	N.S.
+20	0.02±0.09	-0.04±0.02	0.138	N.S.
+40	0.13±0.15	-0.03±0.03	0.034	*
+60	0.24±0.05	0.04±0.03	<0.001	**
+80	0.34±0.08	0.18±0.07	0.002	**
+100	0.52±0.06	0.35±0.04	<0.001	**
+120	0.63±0.09	0.55±0.07	0.099	N.S.
+140	0.72±0.09	0.71±0.06	0.693	N.S.
+160	0.83±0.09	0.85±0.07	0.655	N.S.
+180	0.96±0.00	0.96±0.02	0.579	N.S.

p values were obtained from unpaired t-test. N.S.; not significant, *; $p < 0.05$, **; $p < 0.01$. n = 7 for both constructs.

Table S2. Results of the statistical comparison of $\Delta F/\Delta F_{max}$ values at each membrane voltage between Q507C-TPC3 and Q507C-TPC3 co-expressed with INPP4B

Voltage (mV)	Q507C $\Delta F/\Delta F_{max}$ (±S.D.)	Q507C+INPP4B $\Delta F/\Delta F_{max}$ (±S.D.)	p value	significance
-80	0.11±0.06	0.06±0.02	0.061	N.S.
-60	0.05±0.07	0.06±0.05	0.834	N.S.
-40	0.00±0.03	0.03±0.04	0.216	N.S.
-20	-0.01±0.04	0.01±0.04	0.487	N.S.
0	-0.04±0.04	-0.03±0.03	0.725	N.S.
+20	-0.05±0.04	-0.05±0.02	0.880	N.S.
+40	-0.01±0.03	-0.02±0.04	0.697	N.S.
+60	0.12±0.10	0.02±0.06	0.019	*
+80	0.30±0.06	0.17±0.05	<0.001	**
+100	0.51±0.05	0.37±0.06	<0.001	**
+120	0.65±0.05	0.58±0.05	0.020	*
+140	0.84±0.04	0.75±0.05	0.002	**
+160	0.94±0.05	0.90±0.04	0.101	N.S.
+180	0.99±0.01	0.99±0.02	0.767	N.S.

p values were obtained from unpaired t-test. N.S.; not significant, *; $p < 0.05$, **; $p < 0.01$. n = 8 for Q507C-TPC3, n = 10 for Q507C-TPC3 co-expressed with INPP4B.
